# Supplementary material for: Biomimetic temporal self-assembly via fuel-driven controlled supramolecular polymerization
Source: Nat Commun. 2018 Mar 30;9:1295. doi: 10.1038/s41467-018-03542-z (PMC5878180; doi:10.1038/s41467-018-03542-z)
Supplement: Supplementary file 1 — Supplementary Information [file 41467_2018_3542_MOESM1_ESM.pdf]

Biomimetic Temporal Self-Assembly via Fuel-Driven Controlled Supramolecular Polymerization

Mishra *et. al.*

Supplementary Methods

General: All chemicals were purchased from the commercial sources and were used as such. Spectroscopic grade solvents were used for all optical measurements.

NMR Measurements: NMR spectra were obtained with a Bruker AVANCE 400 (400 MHz) Fourier transform NMR spectrometer with chemical shifts reported in parts per million (ppm) with respect to TMS. Splitting patterns are designated as s, singlet; d, doublet; bs, broad singlet; m, multiplet; t, triplet; dd, doublet of doublet; dt, doublet of triplet.

High Resolution Mass Spectrometry (HRMS): High Resolution Mass Spectra (HRMS) were recorded on an Agilent 6538 Ultra High Definition (UHD) Accurate-Mass Q-TOF-LCMS system using electrospray ionization (ESI) technique either in positive mode or negative mode.

Matrix-Assisted Laser Desorption Ionization (MALDI): MALDI was performed on a Bruker daltonics Autoflex Speed MALDI TOF System (GT0263G201) spectrometer using α-Cyano-4-hydroxy-cinnamic acid (CCA) and sinapic acid (SA) as the matrix.

Spectroscopic Measurements: Electronic absorption spectra were recorded on a Perkin Elmer Lambda 900 UV-Vis-NIR Spectrometer and emission spectra were recorded on Perkin Elmer Ls 55 Luminescence Spectrometer. UV-Vis and emission spectra were recorded in 10 mm path length cuvettes. Circular Dichroism measurements were performed on a Jasco J-815 spectrometer where the sensitivity, time constant and scan rate were chosen appropriately. Corresponding temperature dependent measurements were performed with a CDF – 426S/15 Peltier-type temperature controller. The samples were measured in a 10 mm quartz cuvette.

Dynamic light scattering Experiments (DLS): The measurements were carried out using a NanoZS (Malvern UK) employing a 635 nm laser at a back scattering angle of 173°. The samples were measured in a 10 mm glass cuvette.

Transmission Electron Microscopy (TEM): TEM measurements were performed on a JEOL, JEM 3010 operated at 300 kV. Samples were prepared by placing a drop of the solution on carbon coated copper grids followed by drying at room temperature. The images were recorded with an operating voltage 300 kV. In order to get a better contrast sample was stained with uranyl acetate (1 wt % in water) before the measurements. For TEM, water was used instead of aq. HEPES solution to avoid masking of nanostructures due to HEPES deposition upon drying.

Atomic Force Microscopy (AFM): AFM analyses were carried out on Multimode SPM (Veeco Nanoscope V). Samples were prepared by drop casting 2 µL solution of the sample on a freshly cleaved silicon and HOPG surface and dried under air. Imaging was done under ambient conditions in tapping mode. The probe used for imaging was antimony doped silicon cantilever with a resonant frequency of 300 kHz and spring constant of 40 Nm^{-1}. Software used for analysis of the nanostructures was Nanoscope V7.30r1sr3.

Synthesis and characterization

Precursor compounds **S1**[1], **S2**[2] and **S4**[3] were synthesized based on the reported procedure.

Synthesis of compound S3

S1 (853 mg, 3.7 mmol) and **S2** (1 g, 4.1 mmol) were taken in a flask to which toluene (60 mL) and dry triethylamine (0.43 mL, 2.8 mmol) were added. The reaction mixture was refluxed by using Dean-Stark apparatus for 24 hours and then filtered. The solvent was evaporated and residue was purified with silica gel column chromatography eluting with $CHCl_3$ to get the product as brown pasty material. Yield (88 %,1.5 g). ^{1}H NMR (400MHz, $CDCl_3$, TMS): δ ppm 8.40 (m, 2H), 7.88 (d, 1H, J = 1.2 Hz), 7.81 (dd, 1H, J = 7.8, 1.8 Hz), 7.62 (d, 1H, J = 7.6 Hz), 7.43 (dt, 2H, J = 7.6, 2 Hz), 7.29 (d, 2H, J = 8 Hz), 7.05 (m, 2H), 3.83 (s, 4H), 3.78 (t, 2H, J = 6 Hz), 2.83 (t, 2H, J = 6 Hz). **HR-MS (ESI):** m/z: calculated for $C_{22}H_{19}N_4O_2Br$: 450.0686 $[M]^+$, found: 451.0760 $[M+H]^+$.

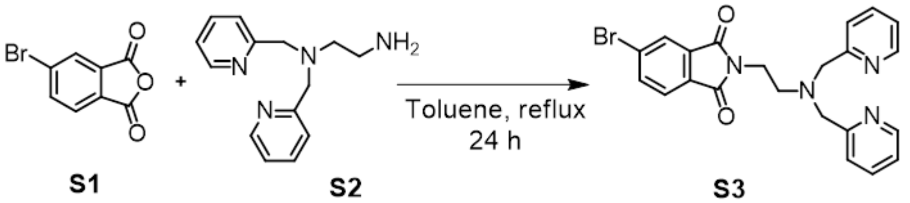

Supplementary Figure 1| Preparation of molecule S3.

Synthesis of compound S5

To **S4** (210 mg, 1.1 mmol), compound **S3** (1.2 g, 2.6 mmol), $Pd(OAC)_2$ (20 mg, 0.08 mmol), tri(*o*-tolyl)phosphine (108 mg, 0.35 mmol), dry DMF (20 ml), and dry triethylamine (1.2 mL) were added and the reaction mixture was heated at 90 ºC for 48 hours. Then DMF was removed and the residue obtained was solubilised in tetrahydrofuran and filtered through celite. Filtrate was dried over Na_2SO_4 and solvent was evaporated. Compound was purified by column chromatography ($CHCl_3$/MeOH, 95/5, *v/v*) followed by SX_3 biobeads ($CHCl_3$) to get the desired product **S5** as yellow solid. Yield of compound **S5** is (11 %, 110 mg).

Note: When compound **S3** was taken in 2.1 equiv., we observed the mono-coupled product as the major product and di-coupled product was in negligible amount. When it was taken in excess of 3 equiv. we observed di-coupled product in considerable amount. ^{1}H NMR (400MHz, $CDCl_3$, TMS): δ ppm 8.44 (m, 4H), 8.02 (s, 2H), 7.81-7.76 (m, 4H), 7.70 (d, 2H, J = 16.4 Hz), 7.45 (dt, 4H, J = 7.6 Hz, 1.8 Hz), 7.36 (d, 4H, J = 8 Hz), 7.25 (d, 2H, J = 16.8 Hz), 7.16 (s, 2H), 7.07 (m, 4H), 3.98 (s, 6H), 3.87 (s, 8H), 3.83 (t, 4H, J = 6 Hz), 2.87 (t, 4H, J = 6 Hz). ^{13}C NMR (100MHz, $CDCl_3$, TMS): δ ppm 168.30, 168.03, 159.34, 152.11, 148.98, 144.10, 136.52, 133.28, 132.10, 130.52, 127.65, 127.38, 126.68, 123.72, 123.27, 122.15, 120.45, 109.53, 60.30, 56.43, 51.87, 36.31. **HR-MS (ESI):** m/z: calculated for $C_{56}H_{50}N_8O_6$: 930.3853 $[M]^+$, found: 931.3941 $[M+H]^+$. **MS (MALDI-TOF) (alpha-cyano-4-hydroxycinnamic acid):** m/z: calculated for $C_{56}H_{50}N_8O_6$: 930.3853 $[M]^+$, found: 931.66 [M+H].

Synthesis of 1

Compound **S5** (50 mg, 0.05 mmol) was dissolved in $CHCl_3$ (5 mL). $Zn(ClO_4)_2{\cdot}6H_2O$ (44 mg, 0.11 mmol) was dissolved in CH_3CN (2 mL), which was added drop wise to the above mixture and stirred at room temperature for 2 hours. The product precipitated in the reaction mixture, which was filtered and washed with $CHCl_3$ and dried to get the desired product **1** as red solid. Yield (89%, 70 mg). ^{1}H NMR (400MHz, CD_3CN, TMS): δ ppm 8.72 (d, 4H J = 5.2 Hz), 8.15 (dt, 4H, J = 7.6, 1.6 Hz), 7.99 (s, 2H), 7.91 (d, 2H, J = 7.6 Hz), 7.78 (d, 2H, J = 7.6 Hz), 7.70-7.63 (m, 10H), 7.46 (d, 2H, J = 16.4 Hz), 7.31 (s, 2H), 4.48 (d, 4H, J = 16.4 Hz), 4.18 (d, 4H, J = 16.4 Hz), 3.94 (s, 6H), 3.88 (t, 4H, J = 7.2 Hz), 2.98 (t, 4H, J = 7.4 Hz). ^{13}C NMR (100MHz, CD_3CN, TMS): δ ppm 168.98, 168.76, 155.43, 152.97, 148.87, 145.50, 142.76, 133.96, 133.31, 131.07, 128.79, 128.09, 127.52, 126.42, 125.99, 124.63, 121.30, 111.10, 57.33, 57.03, 52.23, 33.46. **HR-MS (ESI):** m/z: calculated for $C_{56}H_{50}Cl_4N_8O_{22}Zn_2$: 1454.0377 $[M]^+$, found:1355.0815 $[M-ClO_4]^+$. **MS (MALDI-TOF) (Sinapic Acid)**: m/z: calculated for $C_{56}H_{50}Cl_4N_8O_{22}Zn_2$: 1454.0377 $[M]^+$, found:1355.89 $[M-ClO_4]$.

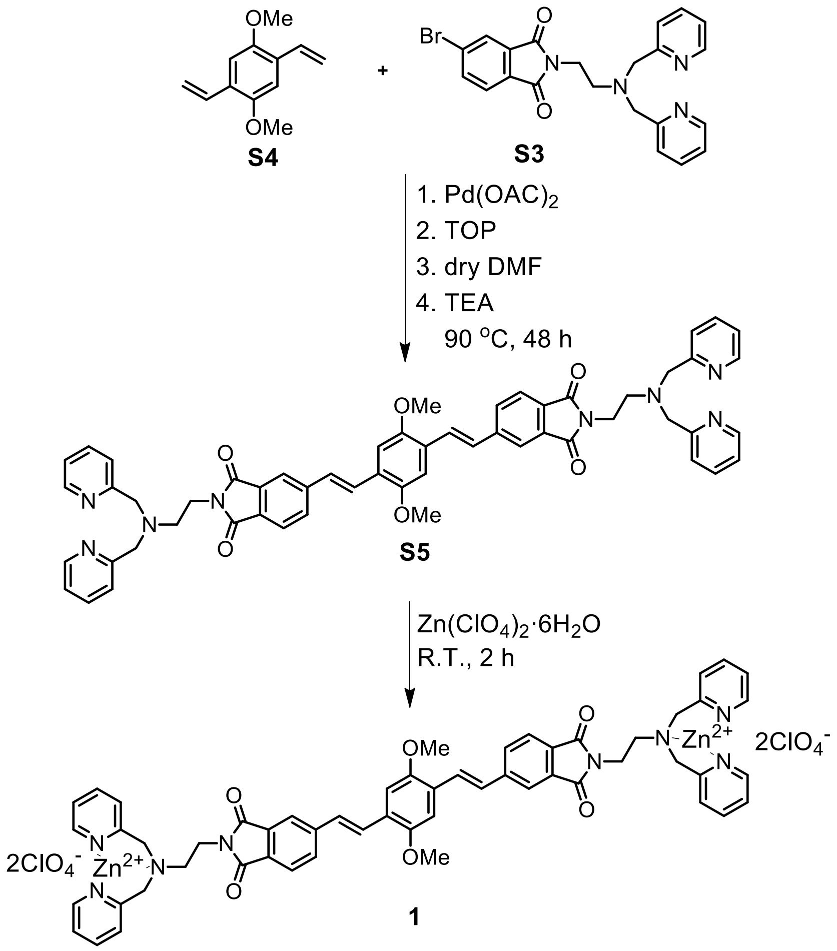

Supplementary Figure 2| Preparation of target molecule **1**.

Synthesis of 2

Compound **S5** (10 mg, 0.01 mmol) was dissolved in $CHCl_3$ (1 mL). $ZnCl_2{\cdot}4H_2O$ (4 mg, 0.02 mmol) was dissolved in CH_3CN (0.2 mL), and added drop wise to the above mixture with continuous stirring at room temperature for 2 hours. The product precipitated in the reaction mixture, which was filtered and washed with $CHCl_3$ and dried to get the desired product **2** as red solid. Yield (75%, 10 mg). ^{1}H NMR (400MHz, DMSO D_6, TMS): δ ppm 8.51 (d, 4H J = 4.4 Hz), 8.2 (m, 4H,), 8.05 (s, 2H), 7.99 (d, 2H, J = 7.2 Hz), 7.86-7.61 (m, 12H), 7.49 (d, 2H, J = 16.4 Hz), 7.46 (s, 2H), 4.59 (d, 8H, J = 16.4 Hz), 3.97 (s, 6H), 3.86 (t, 4H, J = 7.2 Hz), 2.87 (t, 4H, J = 7.4 Hz). **HR-MS (ESI):** m/z: calculated for $C_{56}H_{50}Cl_4N_8O_6Zn_2$: 1198.1190 $[M]^+$, found:1163.1404 $[M-Cl]^+$. **MS (MALDI-TOF) (Dihydroxy Benzoic Acid)**: m/z: calculated for $C_{56}H_{50}Cl_4N_8O_6Zn_2$: 1198.1190 $[M]^+$, found:1163.21 [M-Cl].

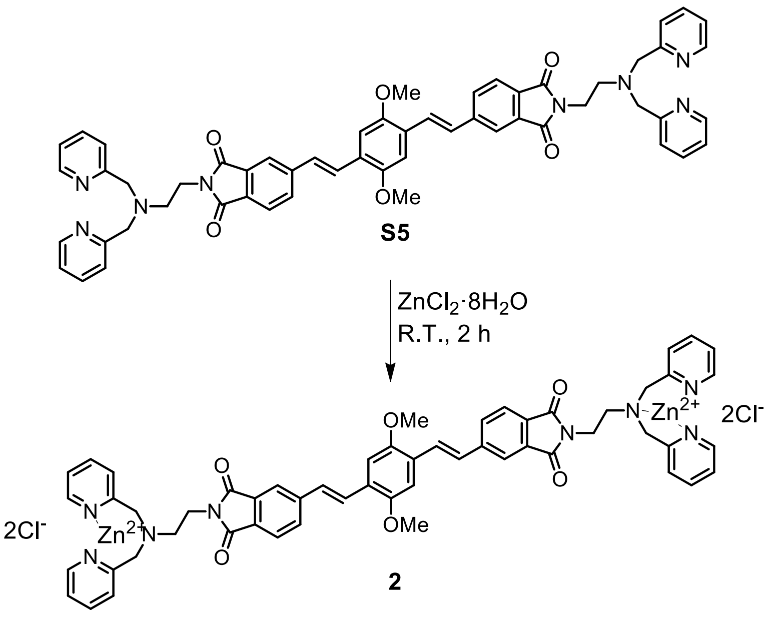

Supplementary Figure 3| Preparation of target molecule **2**.

ATP-induced supramolecular re-organization of 1

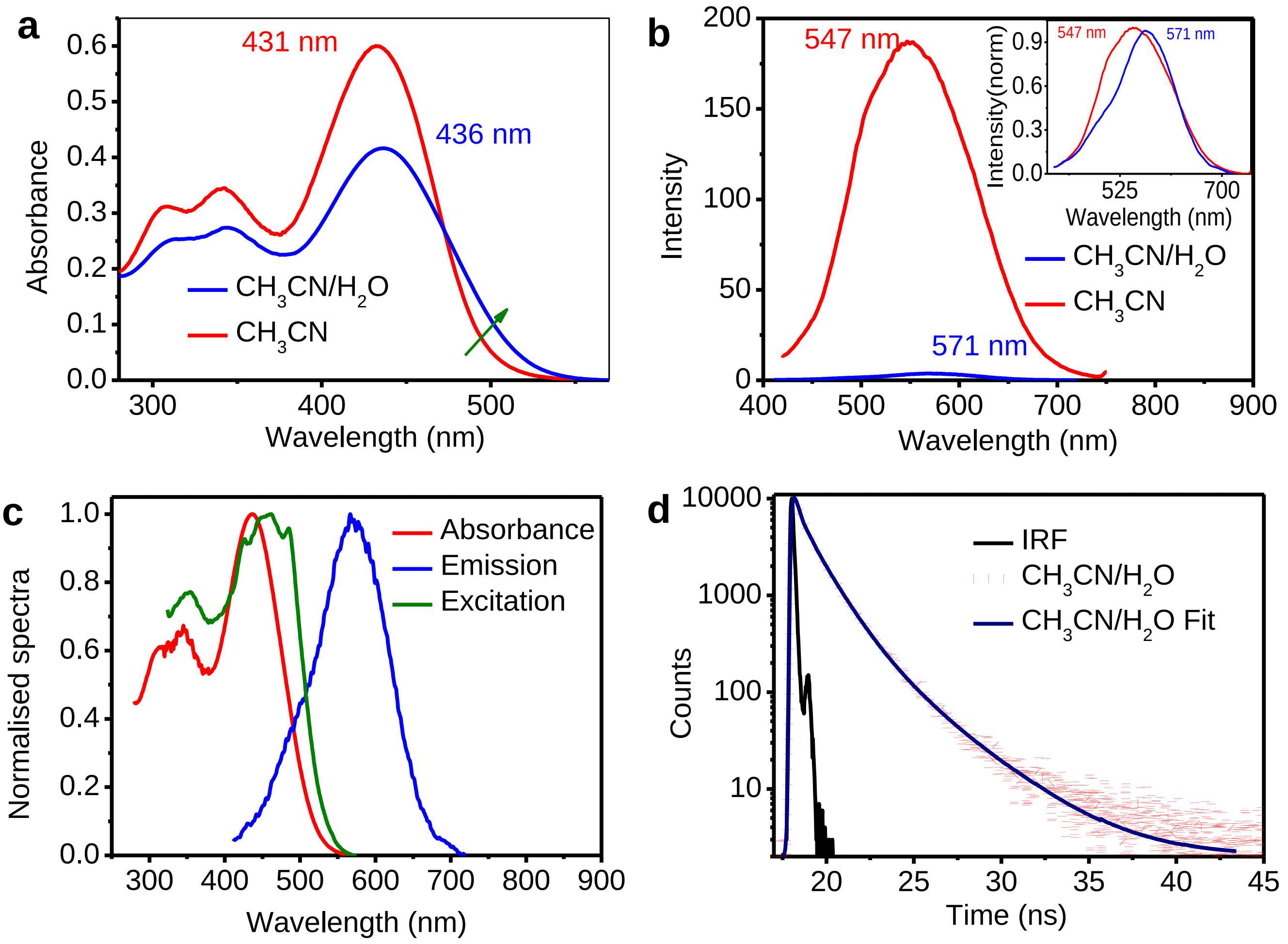

Supplementary Figure 4| Native supramolecular organization of 1. a, Absorption and **b**, emission (λ_{ex} = 385 nm) changes of **1** in CH_3CN/HEPES (100/0, *v/v*) and (10/90, *v/v*) solvent mixtures. Inset of **b**, shows the normalized emission changes. **c**, Normalised absorption, emission and excitation spectra (λ_{em} =570 nm) and **d**, time-resolved fluorescence decay profile ($\lambda_{monitored}$ = 571 nm, λ_{ex} = 375 nm) of **1** in CH_3CN/HEPES (10/90, *v/v*, [**1**] = 2x10^{-5} M, 30 ºC).

Note: Red shift in absorption from 431 nm to 436 nm and emission from 547 nm to 571 nm of **1** on changing composition from CH_3CN to CH_3CN/HEPES mixture suggests the pre-associated nature of the molecule in the presence of water. The red-shifted emission maxima of 571 nm is characteristic of emission as reported for molecules with similar design[4, 5] in a slip-stacked organization due to intermolecular charge-transfer (CT) interactions between the molecules of **1** owing to its A-D-A electronic structure (See Supplementary Fig. 5). The presence of these slip-stacked aggregates is also evident from the excitation spectra which indeed showed a red-shifted maximum at 463 nm compared to the absorption maxima at 436 nm. Fluorescence life-time measurements showed a tri-exponential decay with lifetimes of 0.28 ns (18%), 1.29 ns (64%) and 3.16 ns (18%) for the pre-organized assembly.

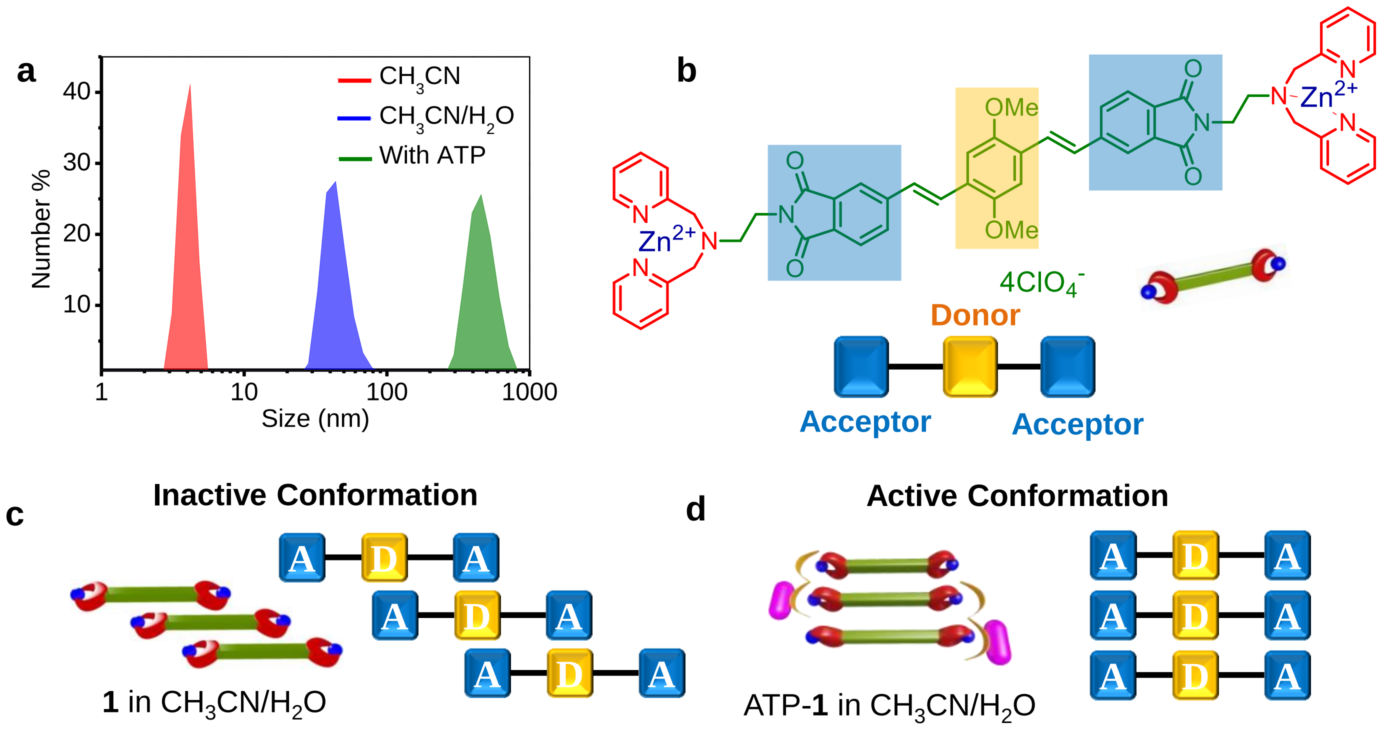

Supplementary Figure 5| ATP-induced switching in supramolecular conformation. **a**, DLS spectra depicting the size of aggregates of **1** in CH_3CN (red), CH_3CN/HEPES (10/90, *v/v*, blue) and **1** with 0.9 equiv. of ATP after elongation (t = 25 min, green) ([**1**] = $2x10^{-5}$ M, 30 ºC). **b**, Molecular structure of **1** and schematic depicting its A-D-A electronic configuration, **c**, schematic showing inactive slipped supramolecular conformation of **1** in the absence of ATP and d, schematic showing active supramolecular conformation of **1** in presence of multivalent fuel, ATP.

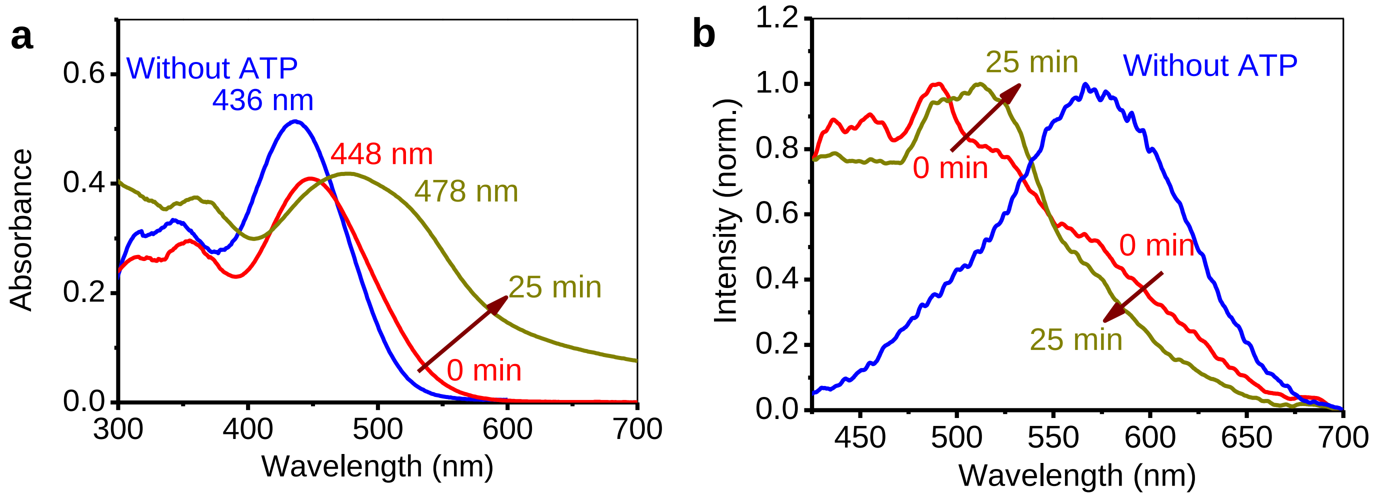

Supplementary Figure 6| Spectroscopic probing of fuel-driven switching in supramolecular conformation. a, Absorption and **b**, emission spectra of **1** in CH_3CN/HEPES (10/90, *v/v*, [**1**] = $2x10^{-5}$ M, 30 ºC) and with 0.9 equiv. of ATP at t = 0 and t = 25 min.

Note. These spectra show immediate red shift in absorbance along with broadening on interaction with ATP from 436 nm to 448 nm at t = 0 min indicating the instantaneous ATP binding to **1**. Over 25 mins, the spectrum shifts further to 478 nm with increase in scattering. The overall change indicates elongation of the stack over 25 mins. From emission spectra changes, we see that on addition of ATP, the emission corresponding to unbound **1** at 571 nm immediately vanishes to give a new blue shifted band at 490 nm corresponding to ATP bound **1**. There is a slight red shift from 490 nm to 512 nm along with increase in emission over a period of 25 mins. Hence these spectral changes were further monitored to characterize the time-dependent growth on binding with fuel.

ATP-induced self-assembly of 1.

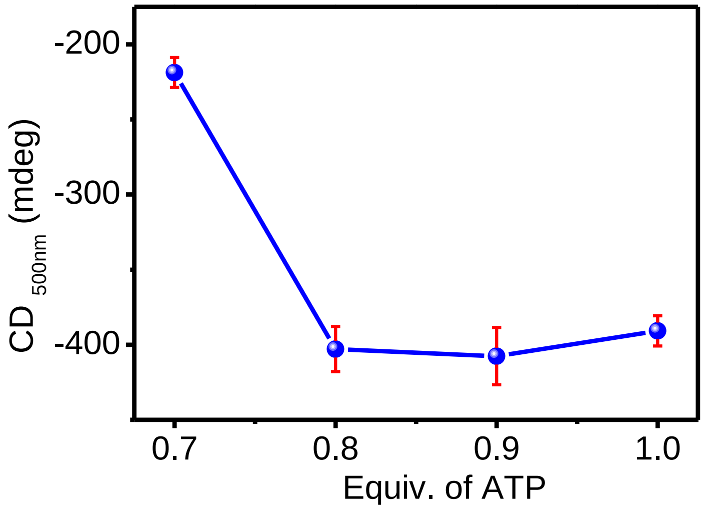

Supplementary Figure 7| ATP Titration of 1. Plot of CD intensity of **1** monitored at 500 nm versus the equiv. of ATP. ([**1**] = 2x10^{-5} M, CH_3CN/HEPES, 10/90, *v/v*, 30 ºC). Error bars have been shown in red for data achieved from experiments carried out for a maximum of 3 times. **Note:** As CD intensity is proportional to the amount of fuel bound, the titration suggests that 0.9 equiv. of ATP is enough to saturate all the binding receptor sites of molecule **1**. Based on this titration, further studies in the present manuscript are performed with 0.9 equiv. of ATP.

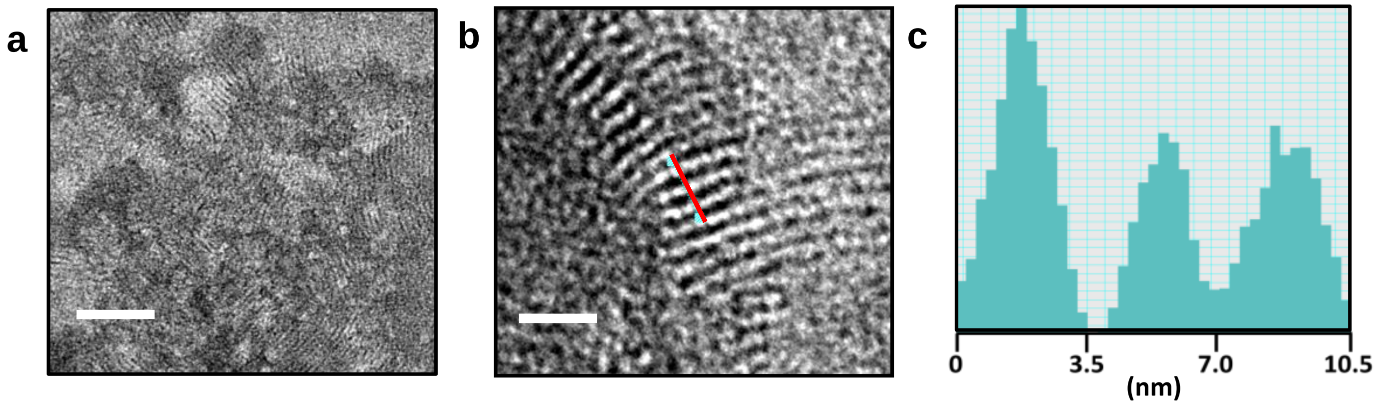

Supplementary Figure 8| TEM showing one-dimensional assemblies of ATP-1. TEM images of **1** with 0.9 equiv. of ATP, stained with uranyl acetate (1 wt% in water). **a**, Scale bar = 80 nm and **b**, zoomed TEM image with scale bar = 30 nm. **c**, Corresponding image histogram (red line in **b**) shows presence of uniform short nanofibres of 3.5 nm, equal to molecular width of ATP-**1** assembly (Supplementary Fig. 10), suggesting that fibres in TEM represent molecular 1-D fibres (CH_3CN/H_2O, 10/90, *v/v*, 0.9 equiv. of ATP, [**1**] = 2x10^{-5} M).

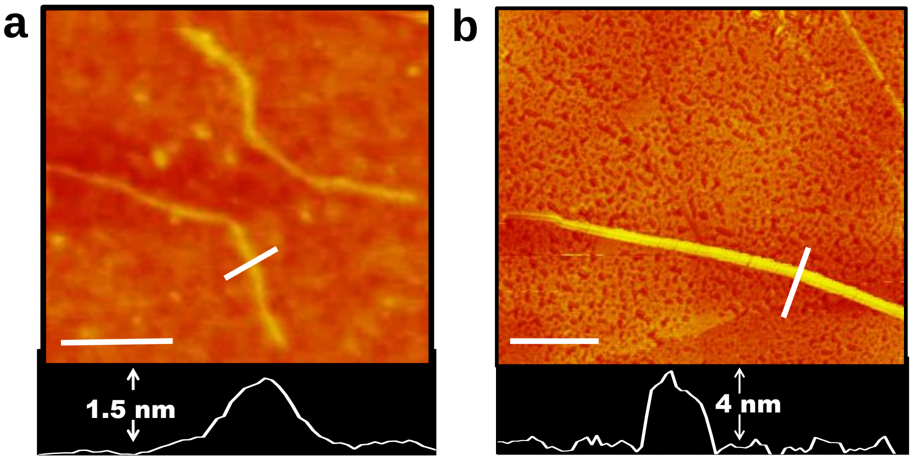

Supplementary Figure 9| AFM analysis ATP-1 assembly. AFM height images of ATP-**1** assembly on **a**, silicon (scale bar, 100 nm) and **b**, HOPG (scale bar, 600 nm) surfaces along with their height profiles. The observed height of the fibre in **a**, is smaller than the calculated width of the ATP-**1** helical stacks (See Supplementary Fig. 11) probably due to the flattening of the fibres on the surface while drying (CH_3CN/H_2O, 10/90, *v/v*, 0.9 equiv. of ATP, [**1**] = $2x10^{-5}$ M).

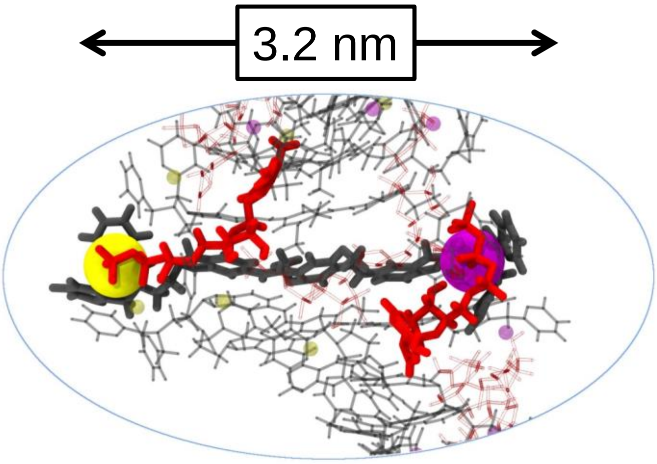

Supplementary Figure 10| Width of ATP-1 helical stacks. Zoomed snapshot of the ATP-**1** helical stacks from the MM/MD simulations at 298.15 K suggesting a width of 3.2 nm for the assembly (Supplementary Fig. 7c and d). Since this dimension is very close to the width of the fibres found from TEM and from the AFM height analyses, we conclude that 1-D fibres are indeed formed by the stacking of ATP-**1** molecules in the growth direction. This structure is extracted from the 25-mer stack. Zinc atoms have been depicted as spheres of two different colours (magenta and yellow) for clarity. Molecules of **1** are depicted as black sticks and ATP is shown in red.

Spectroscopic Probing and Kinetic Analyses of Nucleation-Growth Assembly of 1

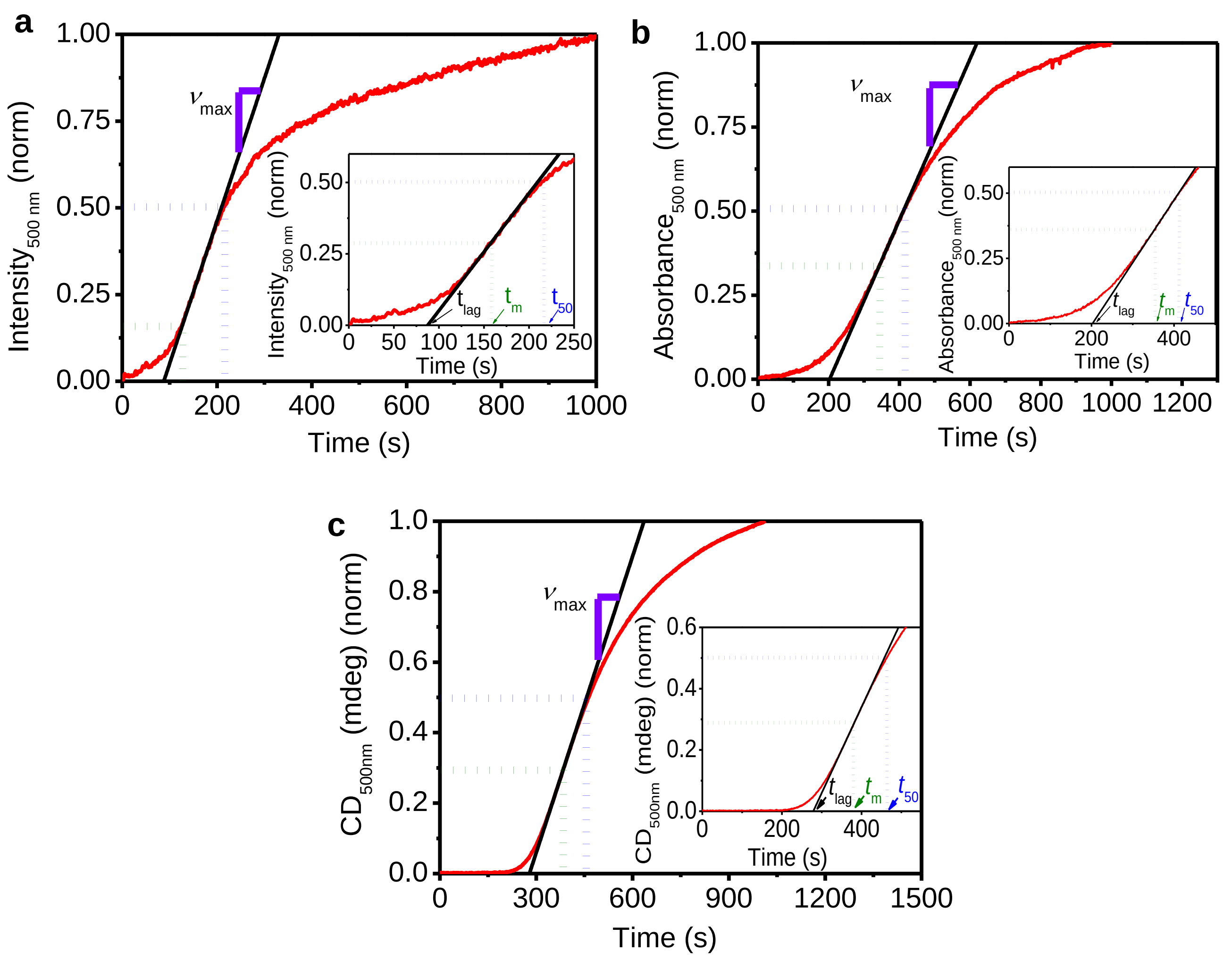

Supplementary Figure 11| Analyses of the elongation parameters with spectroscopic tools. Calculation of t_{lag} (lag time) and t_{50} (half-time i.e. time required for completion of 50% of the process) from tangent drawn at the inflection point t_m (time at which growth rate reaches its maximum i.e. v_{max})[6] using ATP-induced time dependent changes in **a**, fluorescence, **b**, absorbance and **c**, CD of **1** and the obtained values are summarized below in Supplementary Table 1. ([**1**] = 2 x10^{-5} M, CH_3CN/HEPES, 10/90, *v/v*, 0.9 equiv. of ATP, 30 ^{o}C).

Supplementary Table 1| Calculated kinetic parameters of the elongation measured via various spectroscopic tools. Parameters calculated from various spectroscopic changes, t_{lag}, t_m, t_{50} and v_{max}, showed a consecutive increase from fluorescence to absorbance and to CD proving that all these processes do not start at the same time. This indicates that we could monitor various molecular processes through different spectroscopic tools, which lead to the ATP-induced nucleation-growth assembly of **1**.

**Spectroscopic tool**	t_{lag} **(s)**	t_m **(s)**	t_{50} **(s)**	v_{max} **(s^{-1})**
Fluorescence	88 ± 11	160 ± 8	213 ± 4	$4.3 \times 10^{-3} \pm 2.8 \times 10^{-4}$
Absorbance	203 ± 4	375 ± 7	411 ± 2	$2.4 \times 10^{-3} \pm 1.5 \times 10^{-4}$
CD	279 ± 6	388 ± 10	460 ± 7	$2.8 \times 10^{-3} \pm 0.9 \times 10^{-4}$

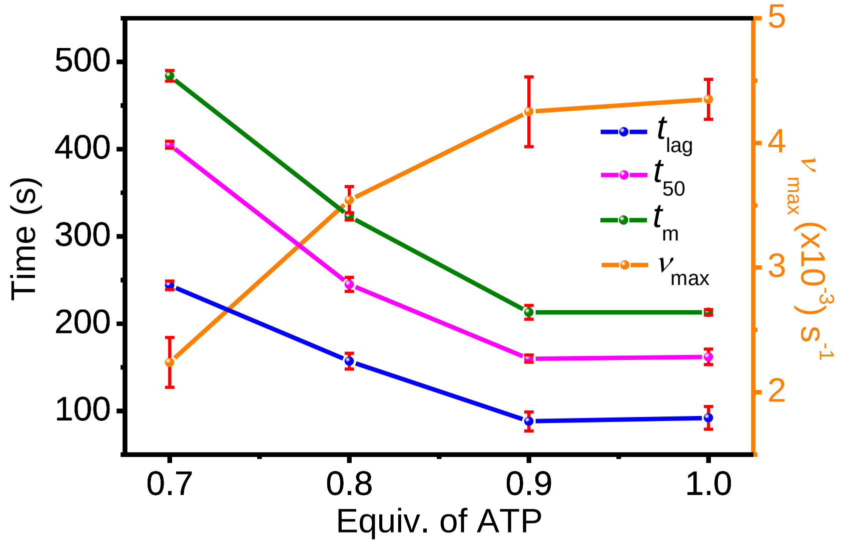

Supplementary Figure 12| ATP Titration of 1. Plot of t_{lag}, t_m, t_{50} and v_{max} from emission changes versus the equiv. of ATP. ([**1**] = 2×10^{-5} M, CH_3CN/HEPES, 10/90, *v/v*, 30 ºC). Error bars have been shown in red for data achieved from experiments carried out for a maximum of 3 times. **Note:** Time-dependent change in the emission is attributed to the allosteric ATP binding induced conformational change of **1** (See Supplementary Fig. 17), which triggers the growth. Hence we further envisage that the saturation of t_{lag}, t_m, t_{50} and v_{max} in the emission changes at 0.9 eq. of ATP further supports this conclusion.

Supplementary Figs. 13-16. To understand the effects of various environmental conditions on the time delayed self-assembly of **1** in presence of ATP, we varied different parameters and witnessed the changes occurring in nucleation-elongation phenomenon under the influence of these parameters

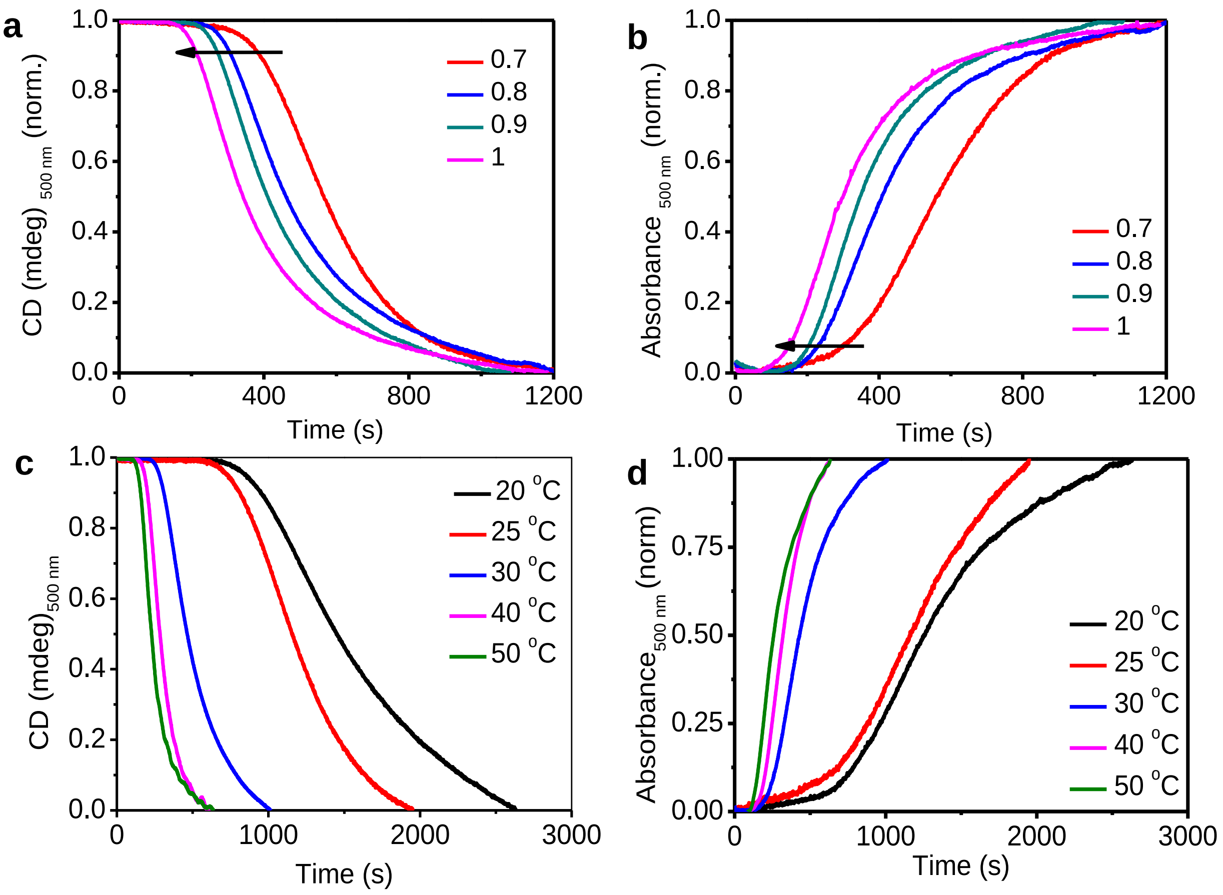

Supplementary Figure 13| Nucleation-growth of ATP-1 assembly with varying environmental conditions. Time dependent changes of **1** in **a**, CD intensity and **b**, absorbance with varying equiv. of ATP at 30 ºC and **c**, CD intensity and **d**, absorbance at different temperatures with 0.9 equiv. of ATP monitored at 500 nm ([**1**] = 2x10^{-5} M, CH_3CN/HEPES, 10/90, *v/v*, 0.9 equiv. of ATP).

Note: With increasing equiv. of ATP there is a decrease in lag phase. This result indicates that the self-assembly kinetics got from absorbance and rearrangement from achiral to chiral kinetics got from CD increases with increasing equiv. of ATP. This also suggests that binding sites are not completely satisfied below 0.9 equiv. of ATP. With increasing temperature there is an increase in the dynamicity of the molecules and hence fastening the process of allosteric binding of ATP and achiral to chiral rearrangement of **1**. Thus there is a decrease in lag phase going from 20 ºC to 50 ºC.

Kinetic fitting of nucleation elongation process. Fitting of the normalized absorbance kinetics of growth to a 2-step model first proposed by Watzky and Finke, which has been used to analyse the self-assembly kinetics of different proteins:[7]

Note: The following equation has been used to fit the data

$$B = \frac{A_o - \frac{\frac{k_n}{k_e} + A_o}{1 + \frac{k_n}{k_e A_o} e^{(k_n + k_e A_o)t}}}{A_o} \quad (1)$$

where A_o is the concentration of the monomeric species that is smaller than the critical-size for nucleation, and B is the concentration of the elongating species that grows exponentially. The model describes two steps: i) nucleation with rate k_n, and ii) growth/elongation with rate k_e.

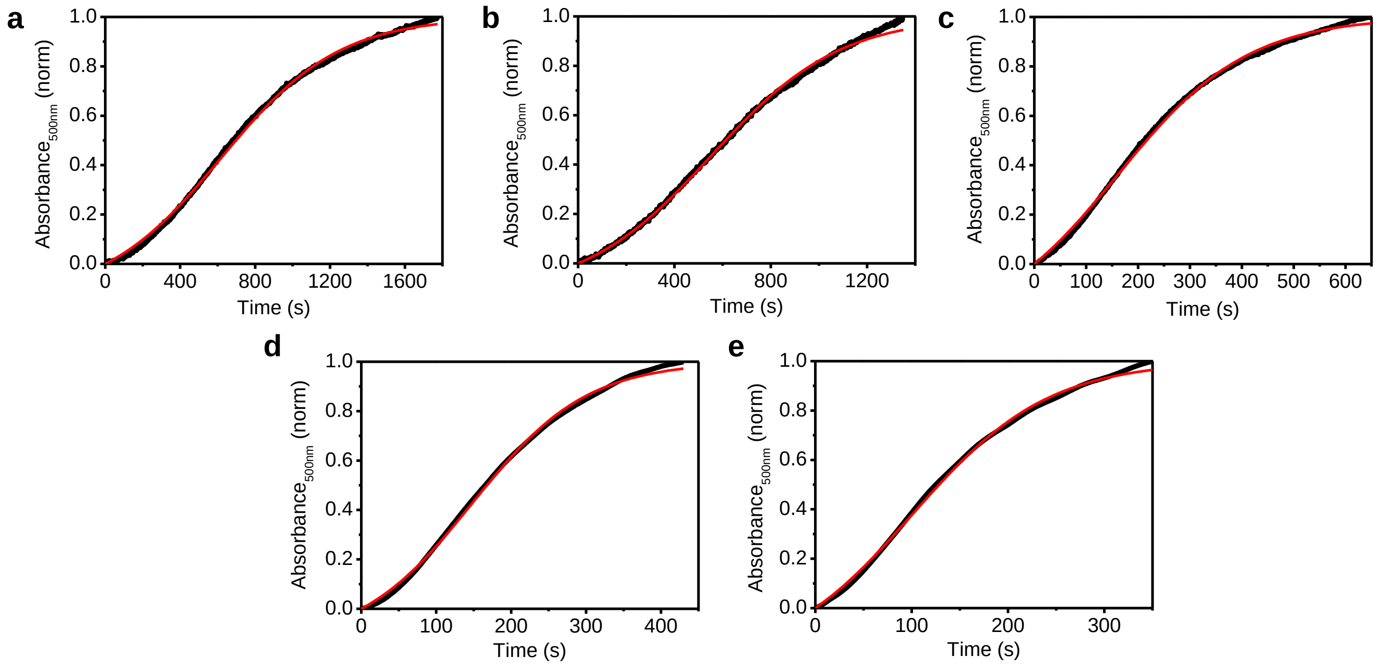

Supplementary Figure 14| Fitting of nucleation-elongation kinetics at different temperatures. Fitting of nucleation-elongation monitored by absorbance using Watzky-Finke equation (Supplementary Equation 1) at **a**, 20 ºC, **b**, 25 ºC, **c**, 30 ºC, **d**, 40 ºC and **e**, 50 ºC.

Supplementary Table 2| Parameters got from fitting of kinetic data in Supplementary Fig. 14. Various parameters derived from the fitted curves at different temperatures by Supplementary Equation 1. There is an increase in k_n as well as k_e with increasing temperature. With increase in temperature the conversion of inactive to active conformation is facilitated, hence fastening the nucleation followed by elongation process. ([**1**] = 2x10^{-5} M, CH_3CN/ HEPES, 10/90, *v/v*, 0.9 equiv. of ATP).

**Temperature**	k_n **(s^{-1})**	k_e **(M^{-1} s^{-1})**	R^2
20 ºC	3.91x10^{-4} ± 2.7x10^{-5}	48.62 ± 0.5	>0.99
25 ºC	4.19x10^{-4} ± 0.5x10^{-5}	58.21 ± 2	>0.99
30 ºC	1.74x10^{-3} ± 1.6x10^{-4}	147.21 ± 0.8	>0.99
40 ºC	1.64x10^{-3} ± 1.0x10^{-4}	200.83 ± 3	>0.99
50 ºC	2.74x10^{-3} ± 2.2x10^{-4}	200.23 ± 1	>0.99

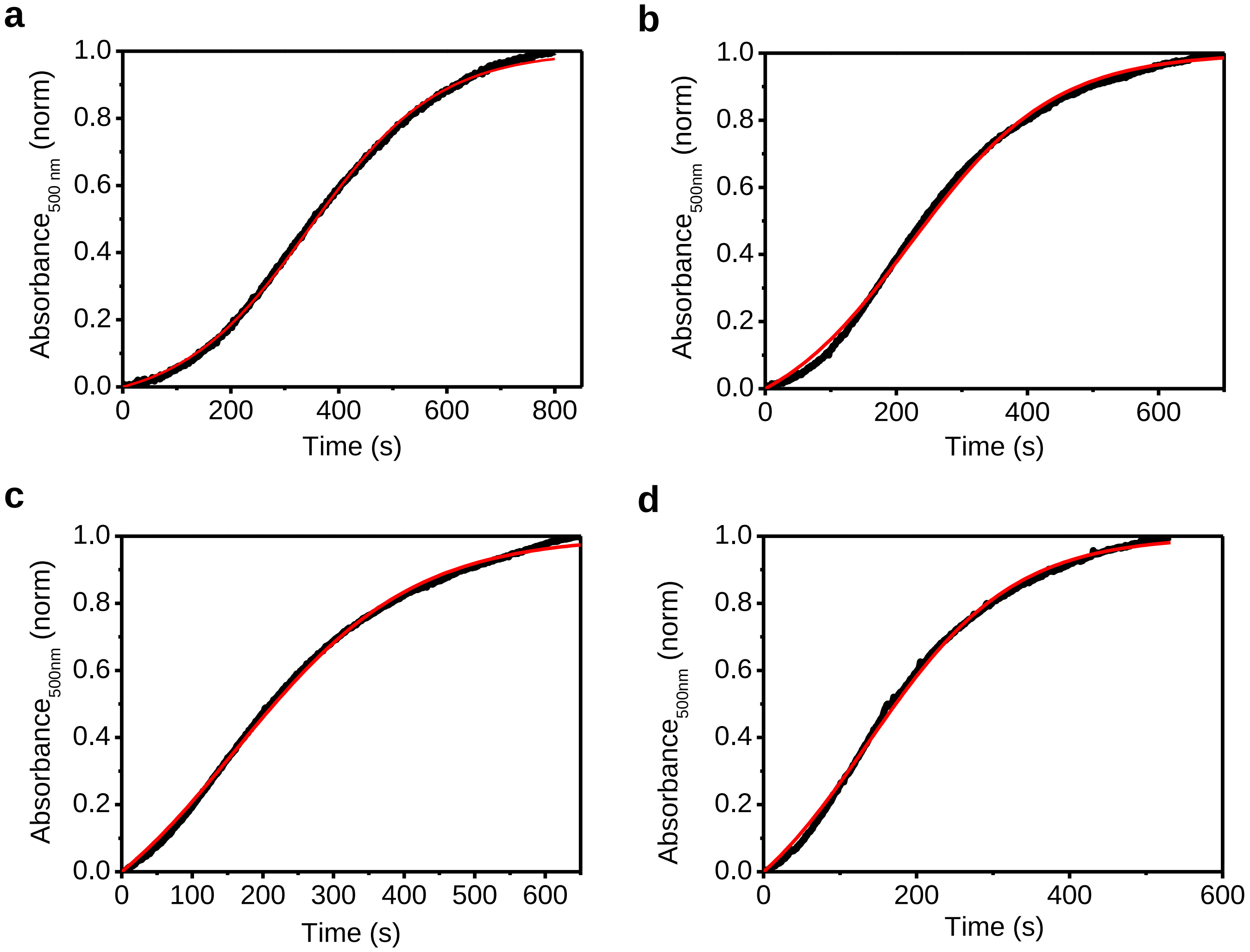

Supplementary Figure 15| Fitting of nucleation elongation kinetics at different equiv. of ATP. Fitting of nucleation elongation growth kinetics (Supplementary Equation 1) monitored at 500 nm by absorbance with **a**, 0.7, **b**, 0.8, **c**, 0.9 and **d**, 1 equiv. of ATP ([**1**] = 2 x10^{-5} M, CH_3CN/HEPES, 10/90, *v/v*).

Supplementary Table 3: Parameters got from fitting of kinetic data in Supplementary Fig. 15. Various parameters derived from the fitted curves by Supplementary Equation 1 at different equiv. of ATP. There is an increase in k_n as well as k_e with increasing equiv. of ATP and saturates at 0.9 equiv. of ATP. ([***1***] = 2x10^{-5} M, CH_3CN/HEPES, 10/90, *v/v*). Equiv. of ATP determines the amount of nuclei giving rise to nucleation followed by elongation. So, with increasing equiv. of ATP, there is an increase in concentration of active nuclei, which facilitates elongation.

**ATP Equiv.**	**k_n (s^{-1})**	**k_e (M^{-1} s^{-1})**	**R^2**
0.7	$4.39 \times 10^{-4} \pm 3 \times 10^{-5}$	139.2 ± 3.5	>0.99
0.8	$1.05 \times 10^{-3} \pm 2.1 \times 10^{-4}$	142.48 ± 2.1	>0.99
0.9	$1.74 \times 10^{-3} \pm 1.6 \times 10^{-4}$	147.21 ± 0.8	>0.99
1	$2.04 \times 10^{-3} \pm 0.9 \times 10^{-4}$	148.59 ± 2.6	>0.99

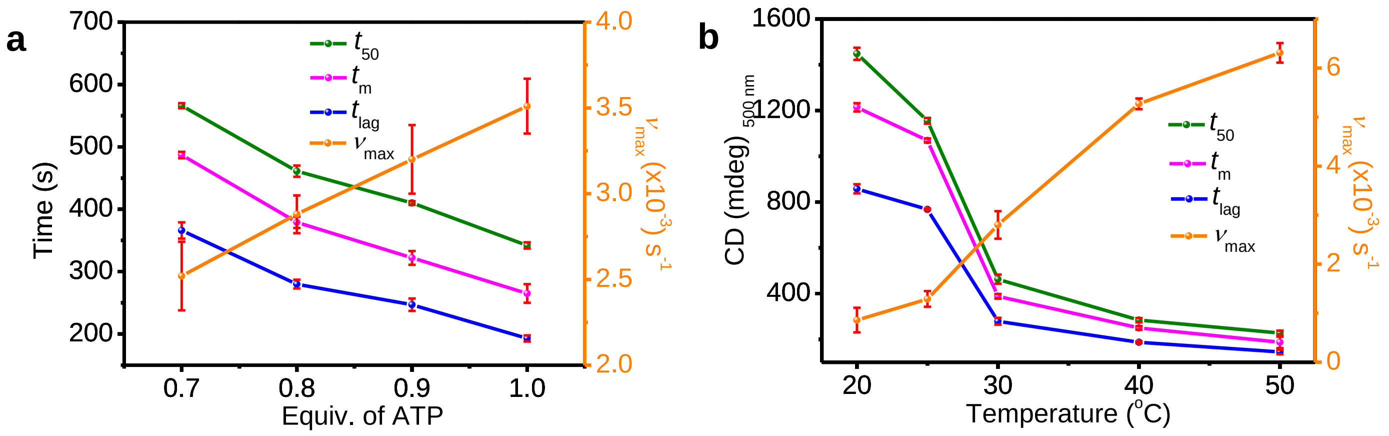

Supplementary Figure 16| Parameters achieved from tangent drawn at inflection point of nucleation elongation. Comparison between t_{lag}, t_m, t_{50} and v_{max} derived from the tangent drawn at inflection point t_m of time dependant absorbance change monitored at 500 nm from the nucleation-elongation curve at **a**, different equiv. of ATP (0.7, 0.8, 0.9 1.0) and **b**, different temperatures (20 ºC, 25 ºC, 30 ºC, 40 ºC, 50 ºC) ([**1**] = 2x10^{-5} M, CH_3CN/HEPES, 10/90, *v/v*). The trends obtained from nucleation elongation kinetics, is reflected in t_{lag}, t_m, t_{50} and v_{max} values. Error bars have been shown in red for data achieved from experiments carried out for a maximum of 3 times.

Allosteric Binding Mechanism of ATP-Binding

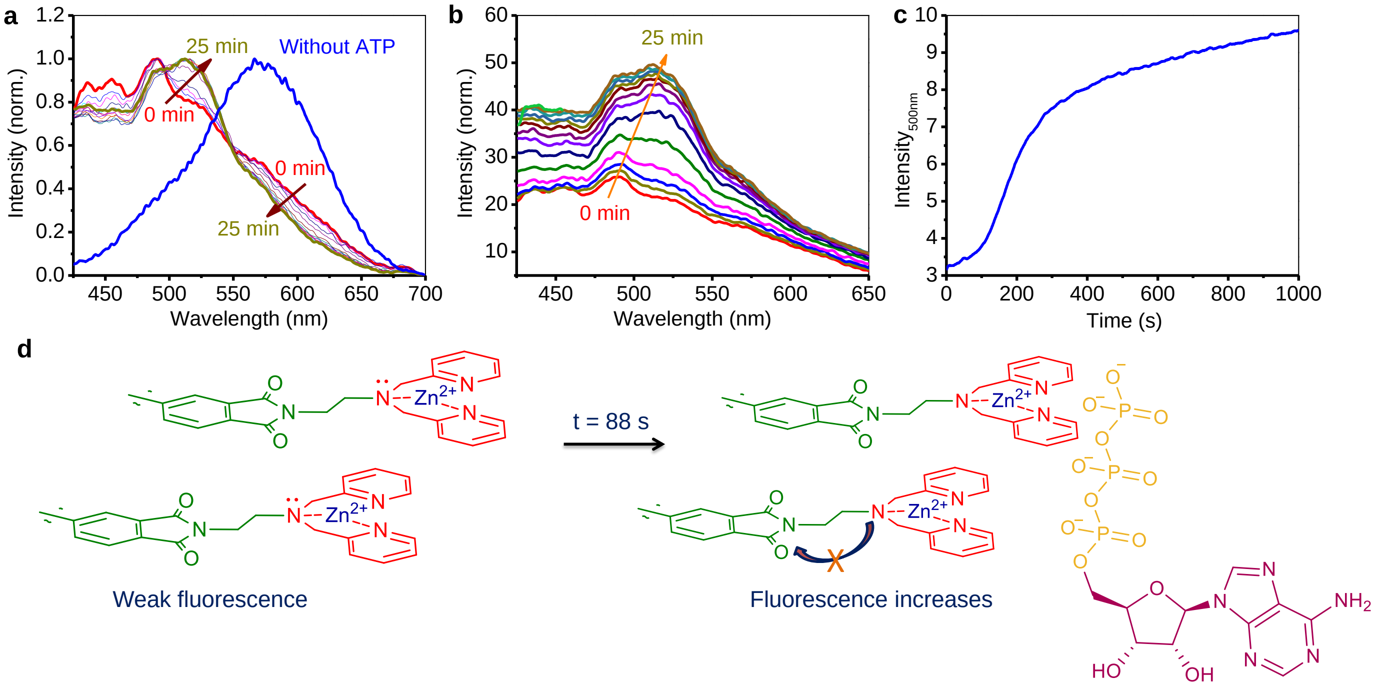

Supplementary Figure 17| Allosteric binding probed via fluorescence. a, Changes in time-dependent normalised emission spectra of **1** in CH_3CN/HEPES (10/90, *v/v*) with 0.9 equiv. of ATP along with the emission spectrum without ATP ([**1**] = $2x10^{-5}$ M, 30 ºC, λ_{ex} = 385 nm). Time dependent **b**, emission spectra and **c**, emission intensity monitored at 500 nm of **1** in CH_3CN/HEPES (10/90, *v/v*) with 0.9 equiv. of ATP. **d**, Proposed allosteric binding mechanism of ATP molecules to the slip-stacked chromophores.

Note: As mentioned in Supplementary Fig. 5, **1** exists in a slip-stacked organization due to its A-D-A electronic structure (inactive conformation or dormant conformation) (*vide infra*) which cannot facilitate any one-dimensional growth. However, as soon as ATP is introduced into the solution, an immediate red shift and broadening of absorbance band (431 nm to 436 nm) was observed (see Supplementary Fig. 3) This change was accompanied by an instantaneous blue shift in emission spectra from 571 nm to 490 nm, on addition of ATP, a clear indication of fast binding of the ATP and resultant changes in the molecular packing from a slip-stacked structure.

Subsequent changes in the emission spectra with time, such as the red shift from 490 nm to 512 nm, along with the increase in emission over a period of 25 mins, are indicative of an allosteric binding of ATP with **1**. It has been shown in DPA-Zn based molecular sensors for ATP that Zn^{2+} is very loosely bound to the nitrogen atoms of DPA by weak co-ordinate bonds.[8, 9] As a result, binding of multi-valent phosphate to single receptor site via single phosphate group does not prevent the intramolecular charge-transfer from

DPA to the imide completely, resulting in partially quenched emission. However, it has been shown that binding of the second phosphate group to another pre-organized receptor re-enforces both the binding of the multi-valent phosphate to the receptor site and also the coordination of the Zn^{2+} to the DPA sites. This allosteric binding leads to an increase in emission as it prevents the electron transfer from DPA to the chromophore completely. We envisage that the observed time-dependent cooperative emission increase in our system is indeed because of a similar allosteric binding of the ATP to the multiple DPA sites along the stacking direction. Monitoring the fluorescence at 500 nm over time also showed a sigmoidal increase, characteristic of an allosteric rearrangement. There is a gradual increase with a time lag of 88 s which makes this transformation the first chronological event *en route* to a fully grown chiral stack. Most importantly, the fluorescence increase is almost 70% over by the time increase in absorbance and elongation of the assembly sets in. Since the electrostatic binding of ATP to the molecules of **1** is instantaneous, the observed time-dependent changes in emission indicate to a supramolecular reorganization into an active conformation conducive for nucleated 1-D growth.

MM/MD simulations for ATP-induced self-assembly of 1.

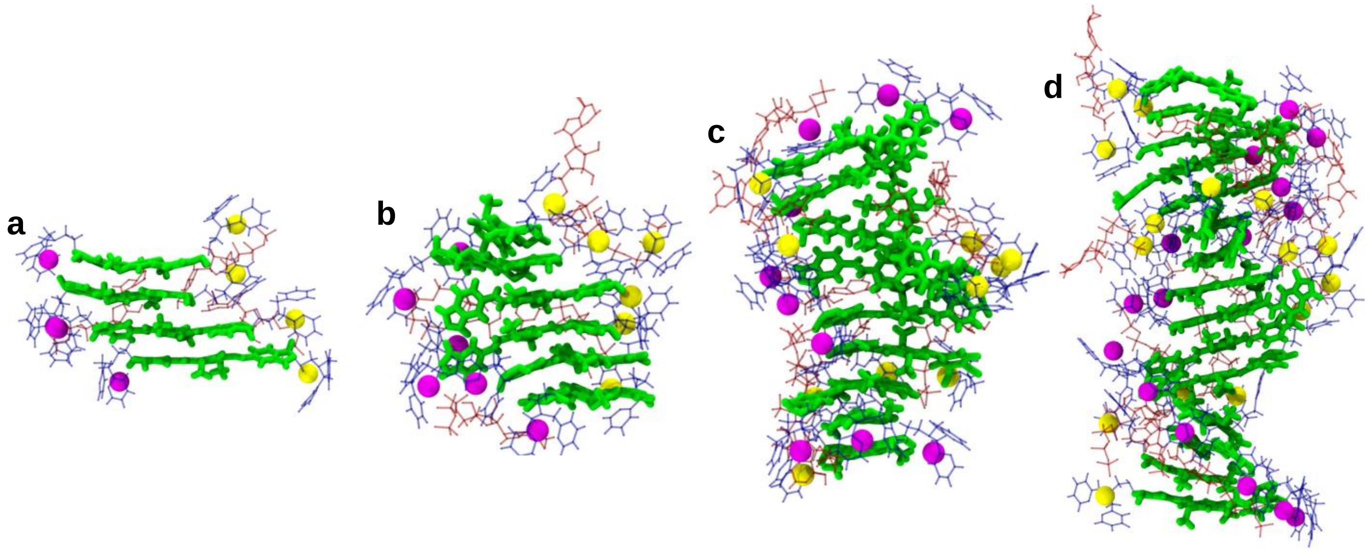

Supplementary Figure 18| MM/MD simulations of ATP-1 oligomers. Snapshots of the simulated oligomers of **a**, tetramer, **b**, hexamer, **c**, decamer and **d**, pentadecamer. (OPV core is depicted by thick green sticks, DPA and ATP have been represented by thin blue and red sticks, Zn atoms are depicted in spheres, with yellow on one side and magenta on the opposite, to clearly bring about the chirality of the assembly). Other oligomers are shown in Fig. 2g of main text.

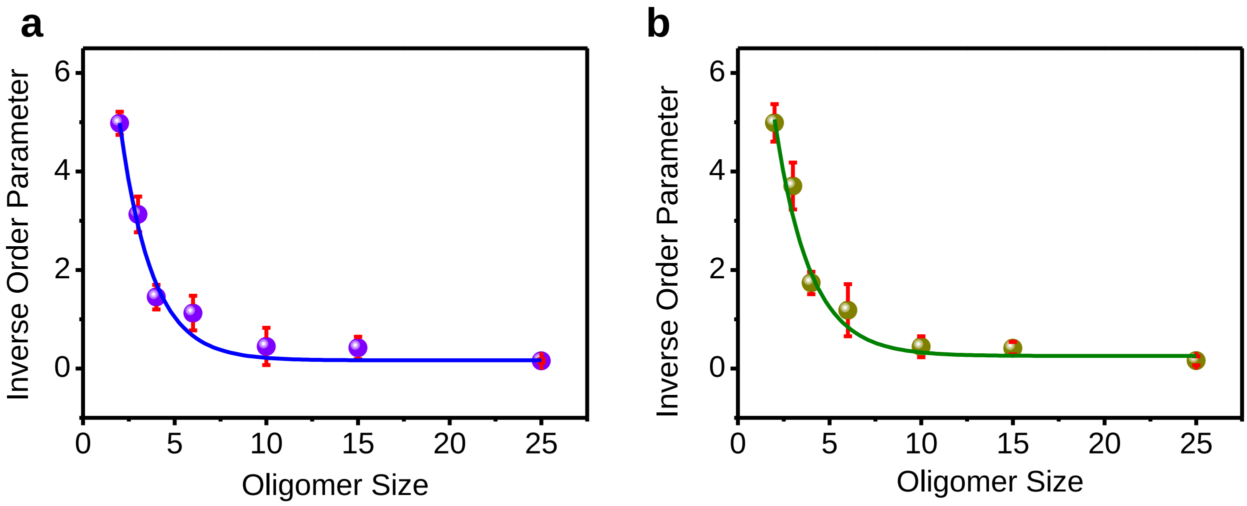

Supplementary Figure 19| Inverse Order Parameter of simulated structures. Inverse Order Parameter (Supplementary Equation 1) of various oligomers extracted from MM/MD simulations of ATP-**1** stacks with reference frame chosen for the calculation of RMSD: **a**, 28 ns and **b**, 29 ns. The same with reference frame chosen as at 30 ns is shown in Fig. 2f of main manuscript. The behaviour of the Inverse Order Parameter (IOP) with oligomer size is independent of the chosen reference frame. The error bars were calculated using the standard error formalism, by considering the RMSD data over time as blocks.

Note. The induced chirality in the oligomers was quantified through an Inverse Order Parameter (IOP) defined as the root mean square displacement (RMSD) per dimer in oligomer.

$$\text{IOP} = \text{RMSD}/((n\text{-}1)^{*}\pi\text{-}\ \pi \text{ distance}) \quad (2)$$

where n is oligomer size and the π- π distance was taken to be 3.8 Å. As mentioned earlier, each oligomer was simulated in solution for 30 ns and the coordinates of the ATP-**1** or GTP-**1** systems were saved every 2.5 ps. Post an equilibration period of 5 ns, the RMSD was calculated for the oligomer in each frame *with respect to* the coordinates of the stack at 30 ns (reference structure). In the calculation of RMSD, only the coordinates of the OPV segments of the stack were used. RMSD, thus calculated, was averaged over all the frames for a given oligomer. Fig. 2f of the main manuscript provides the IOP calculated using the oligomer coordinates at 30 ns as reference. In Supplementary Fig. 20 (a and b)**,** we show the same quantity calculated with either the structure at 28 ns or at 29 ns as reference. The near invariance in the behaviour of IOP (Supplementary Equation 2) confirms that the results are independent of the choice of the reference frame. The IOP decreases with increase in the oligomer size irrespective of the reference structure.

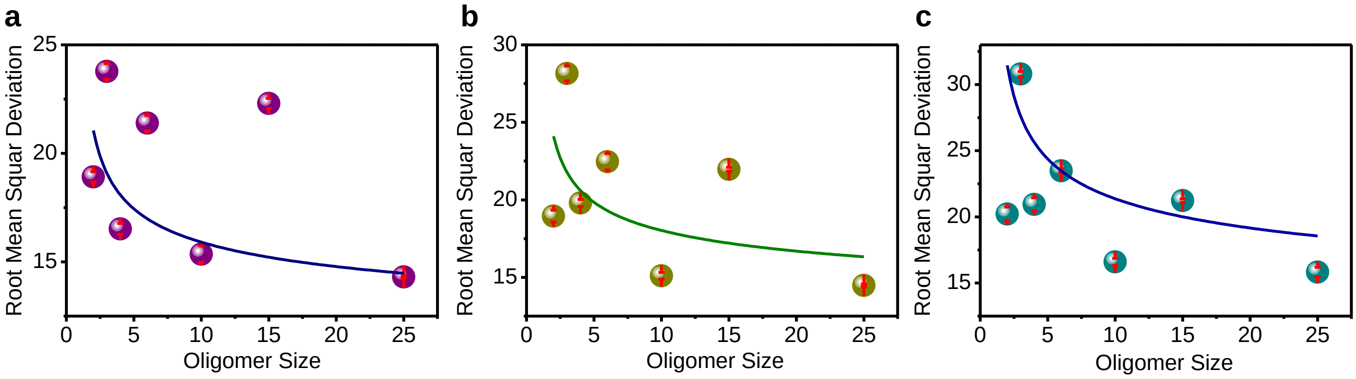

Supplementary Figure 20| Root Mean Square Deviation of various oligomers. RMSD extracted from MM/MD simulations of ATP-**1** stacks with reference frame chosen for the calculation of RMSD: **a**, 28 ns and **b**, 29 ns and **c**, 30 ns. The error bars were calculated using the standard error formalism, by considering the RMSD data over time as blocks.

IOP has been calculated from the RMSD values using the following expression.

$$\text{IOP} = \text{RMSD}/((n\text{-}1)^{*}\pi\text{- } \pi \text{ distance}) \quad (2)$$

The RMSD exhibits a weak decrease with increase in oligomer size which leads to much lower values of IOP. While performing simulation on systems with various oligomer sizes, the initial geometry of all oligomers were constructed with same twist angle (i.e. 25°), which indeed induced helicity in the stack. During the MD simulations, the molecules in shorter oligomers exhibit structural differences with this initial configuration such as slip between the consecutive molecules (discussed in the Supplementary Table 14) and a deviation in the twist angle. To probe these two parameters in all oligomers, we used the RMSD of the oligomer coordinates with respect to arbitrarily chosen, well equilibrated configurations. The RMSD helps us in investigating the deviation of the twist angle as well as the slip distance from such reference structures. The IOP tells us about the arrangement of (consecutive molecules in an oligomer) with respect to a free dimer. In the case a free dimer we observed a very large lateral slip and almost no twist when compared to initial configuration which leads to a high IOP. As molecules in longer stacks exhibit a significant twist angle, their lower values of IOP imply lower fluxionality and greater stability of the stack implying chirality.

ATP-selective co-operative self-assembly

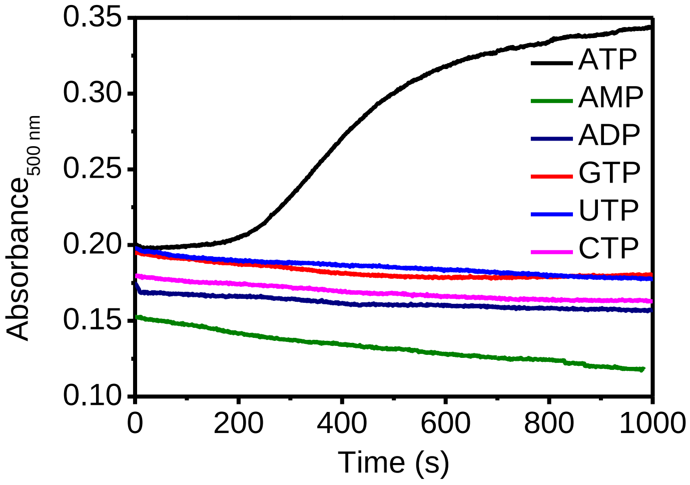

Supplementary Figure 21| ATP-selective growth. Time dependent absorbance changes of **1** at 500 nm on interaction with ATP, AMP, ADP, GTP, UTP and CTP. ([**1**] = 2x10^{-5} M, CH_3CN/HEPES, 10/90, *v/v*, 0.9 equiv. ATP, GTP, UTP and CTP, 2 equiv. of ADP and 5 equiv. of AMP, 30 ºC; absorption spectral changes suggest that the binding sites are indeed saturated with the corresponding fuels at these equiv.). This indicates that only ATP induces elongation of **1** even though the other triphosphates are able to bind to the molecule (See Fig. 3c).

ATP-selective seeded supramolecular polymerization of 1

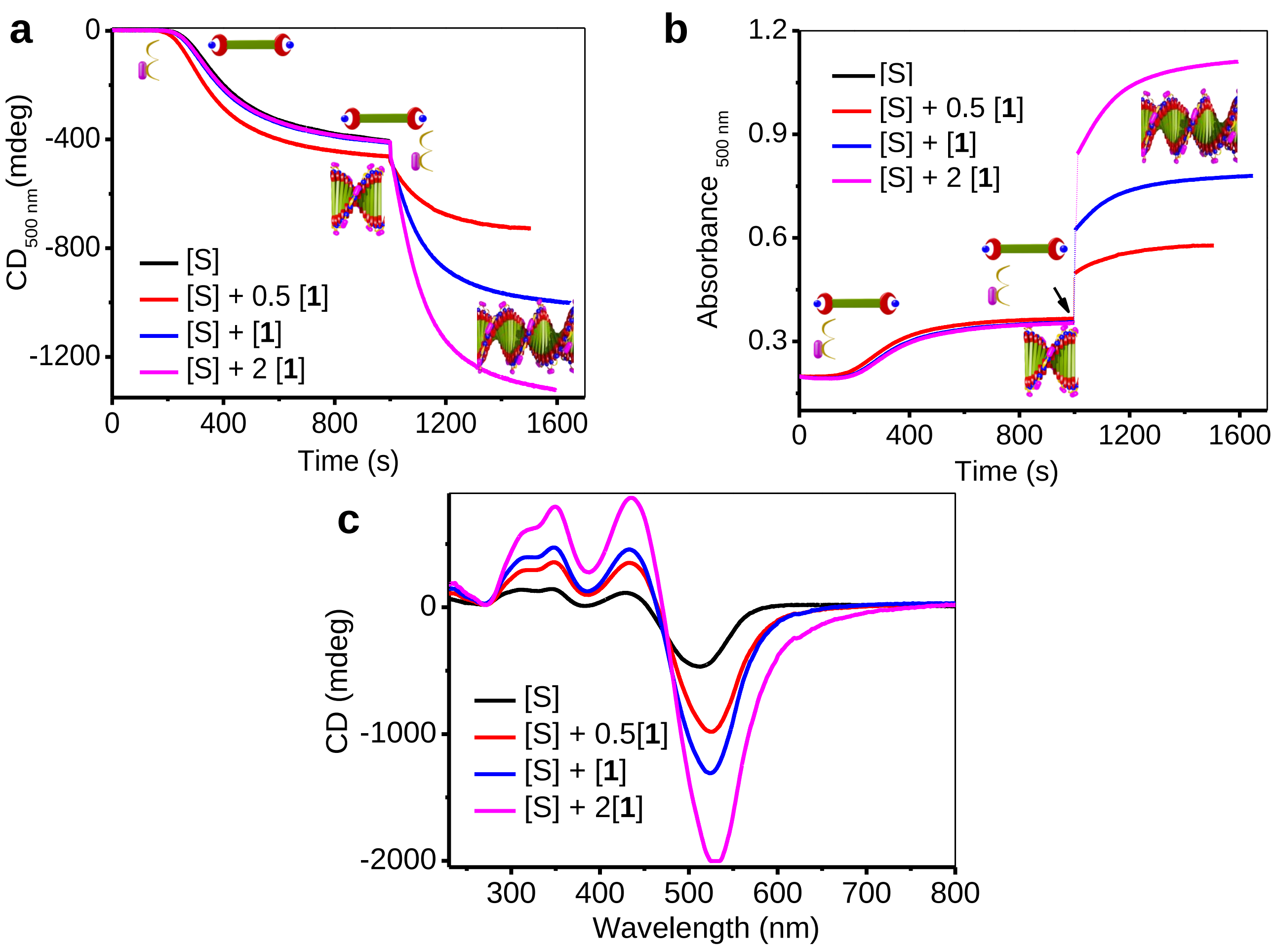

Supplementary Figure 22| Seeded supramolecular growth. Time dependent **a**, CD intensity and **b**, absorbance monitored at 500 nm showing seeded growth for various [S]/[**1**] ratios of 1:0.5, 1:1 and 1:2. **c**, Final CD spectra after seeded polymerization. ([S] = $2x10^{-5}$ M, [**1**] = $1x10^{-5}$ M (10 μL), $2x10^{-5}$ M (20 μL) and $4x10^{-5}$ M (40 μL), CH_3CN/HEPES, 10/90, *v/v*, 0.9 equiv. ATP, 30 ºC). On addition of a fresh feed of **1**, there is an immediate increase in CD signal as well as absorbance with the absence of any lag phase. There is an immediate jump observed in absorbance on addition of fresh feed which corresponds to the increase in optical density due to overall increase in the concentration of **1** in solution.

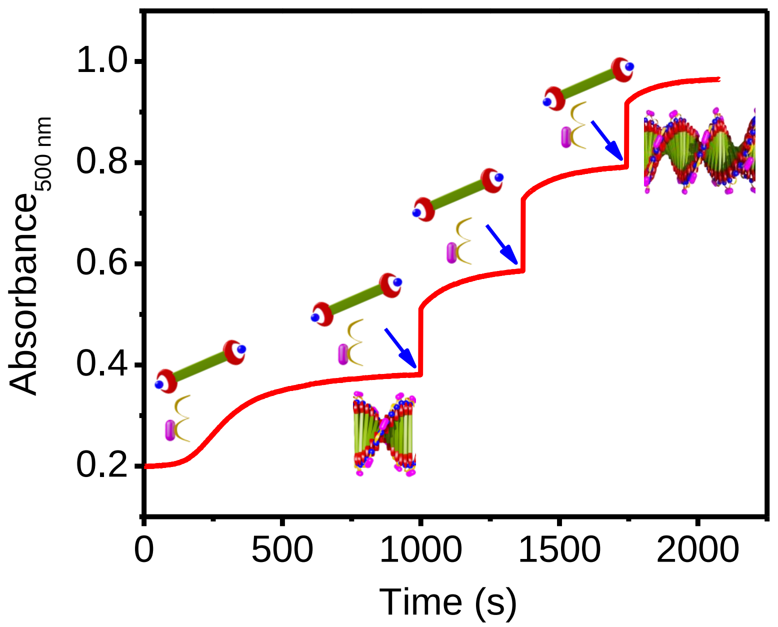

Supplementary Figure 23| Seeded growth of 1 probed by absorbance. Absorbance monitored at 500 nm showing seeded growth on three subsequent addition of fresh feed of **1** with ATP. ([**1**] = 1x10^{-5} M (10 μL), [S] = 2x10^{-5} M, CH_3CN/HEPES, 10/90, *v/v*, 0.9 equiv. ATP, 30 ºC).

Note. On addition of a fresh feed of **1** along with equiv. amount of ATP, there is an immediate increase in absorbance for the three additions due to the increase in optical density of the added molecules of **1**. After an immediate increase in absorbance, a gradual elongation process is evident over time. Absence of lag-phase clearly indicates a seeded growth. Corresponding CD changes are shown in Fig. 4a.

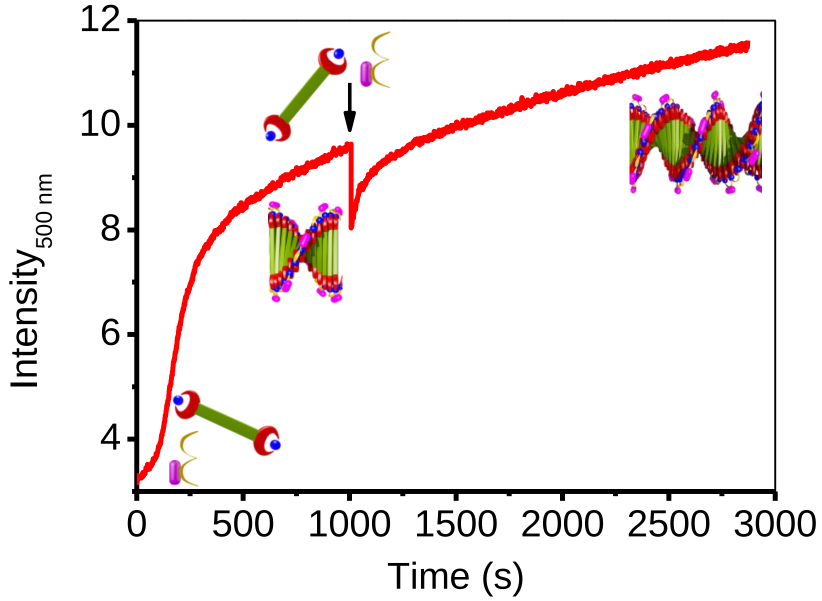

Supplementary Figure 24| Seeded growth of 1 monitored by fluorescence. Emission intensity monitored at 500 nm showing initial allosteric binding induced sigmoidal change in emission and the immediate increase in emission on addition of the fresh feed of **1** with ATP ([S] = $2x10^{-5}$ M, [**1**] = $1x10^{-5}$ M (10 μL), CH_3CN/HEPES, 10/90, *v/v*, 0.9 equiv. ATP, 30 ºC).

Note. On addition of a fresh feed of **1**, there is an increase in emission with the absence of any sigmoidal change in emission features. This suggests the seeding process and a fast conformational change in the fresh feed of monomers on seeded growth. Subsequent increase in the emission is a signature of the growth of the stacks. (The initial dip in the emission on addition of fresh feed is due to self-absorption at high concentrations of **1** as a result of overlapping absorption and emission features).

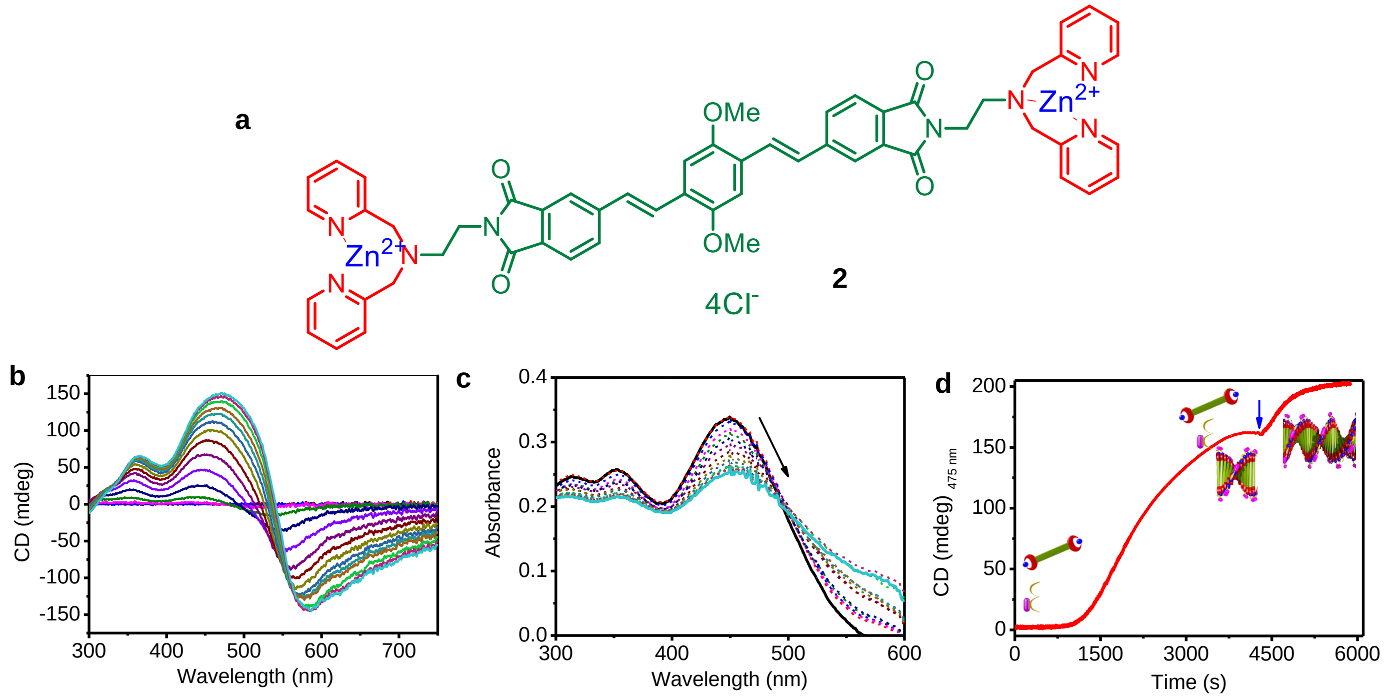

Supplementary Figure 25| Nucleation-elongation followed by seeding for molecule 2. a, Molecular structure of **2**. **b,** CD and **c,** UV-Vis absorbance spectra represent time lapse spectra of ATP-driven growth of **2. d,** CD signal showing seeded supramolecular polymerization of **2** upon a subsequent addition of monomer with ATP ([S] = 2x10^{-5} M, [**2**] = 1x10^{-5} M (10 μL of (5x10^{-3} M stock) and 0.9 μL of ATP (10^{-2} M)) HEPES/CH_3CN, 90/10, *v/v*, 0.9 equiv. ATP, 30 °C). The above results show that even on changing the counter ions of the molecule, the properties do not change.

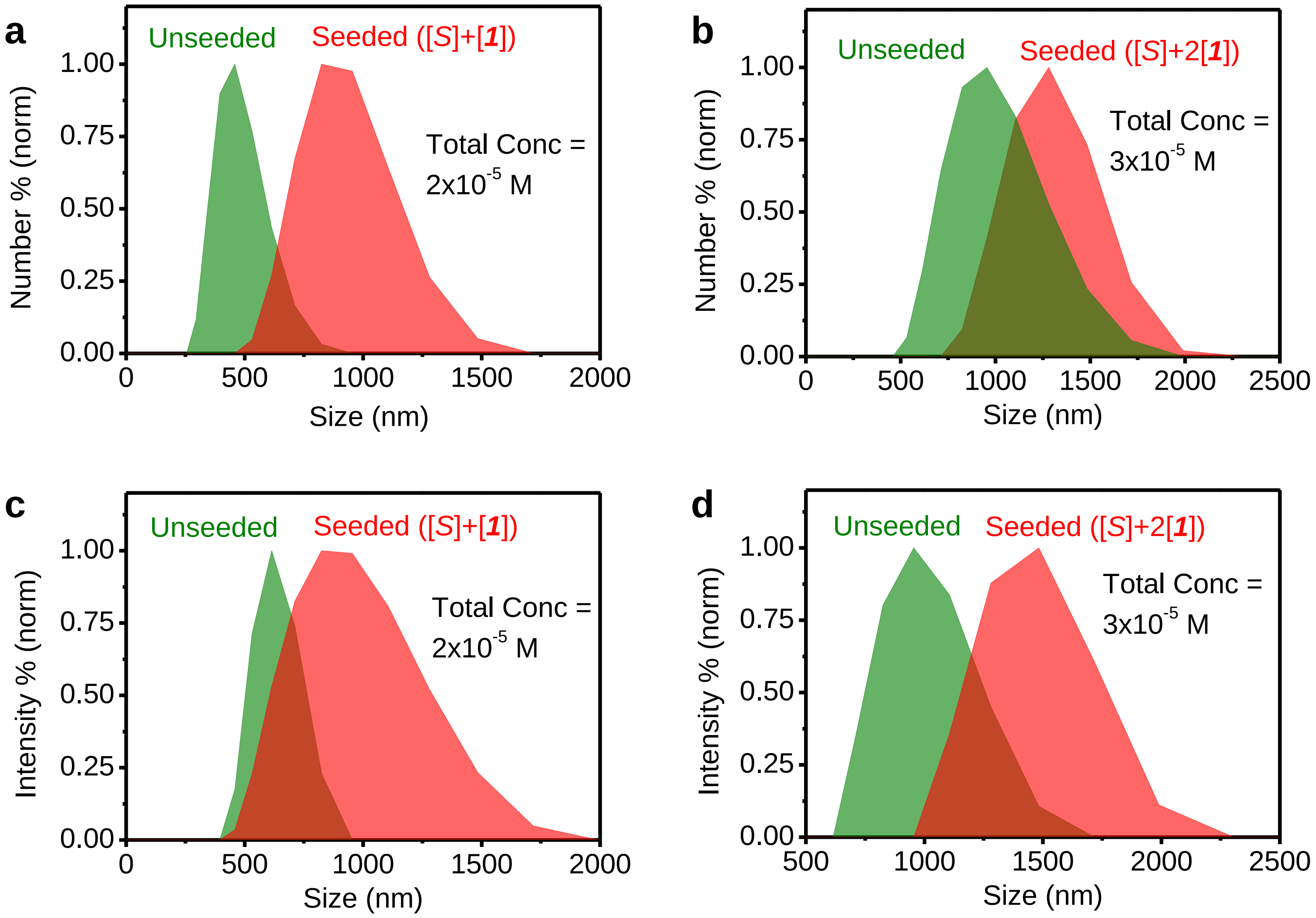

Supplementary Figure 26| Comparison in DLS data for seeded and non-seeded growth. Size of stacks, got from number % (**a** and **b**) and intensity % (**c** and **d**), grown via seeded (red) as well as unseeded (green) where the total concentration was maintained same (total concentration in **a** and **c**, *c* = 2x10^{-5} M and in **b** and **d**, *c* = 3x10^{-5} M). For both the concentrations, the size of the seeded stack is larger than the unseeded stack in consistent with a seeded growth ([S] = 1x10^{-5} M, **a**, [**1**] = 1x10^{-5} M (10 μL) and **b**, [**1**] = 2x10^{-5} M (20 μL), CH_3CN/HEPES, 10/90, *v*/*v*, 0.9 equiv. ATP, 30 ºC).

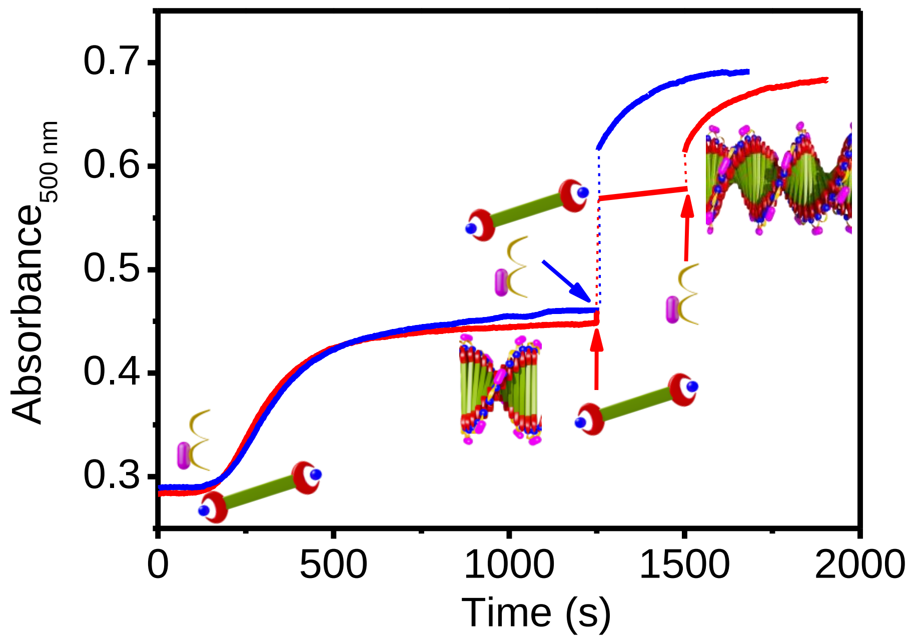

Supplementary Figure 27| ATP-Templated seeded growth. Overlaid curves of absorbance changes with simultaneous (blue) and consecutive (red) addition of **1** and ATP to pre-grown seed. ([S] = 2x10^{-5} M, [**1**] = 1x10^{-5} M (10 µL), CH_3CN/HEPES, 10/90, *v/v*, 30 ºC). To a pre-grown stack of ATP-**1**, when a fresh feed of **1** is added without ATP an immediate rise in absorbance monitored at 500 nm is seen due to the increase in optical density of fresh feed but remains constant after that. However, when ATP is introduced again, absorbance increased indicating the seeded growth. This further shows that the elongation of the stacks can be controlled with the concentration of fuel and a templated growth of the stack is required for the molecule to show seeded polymerization. Corresponding CD changes are shown in Fig. 4b.

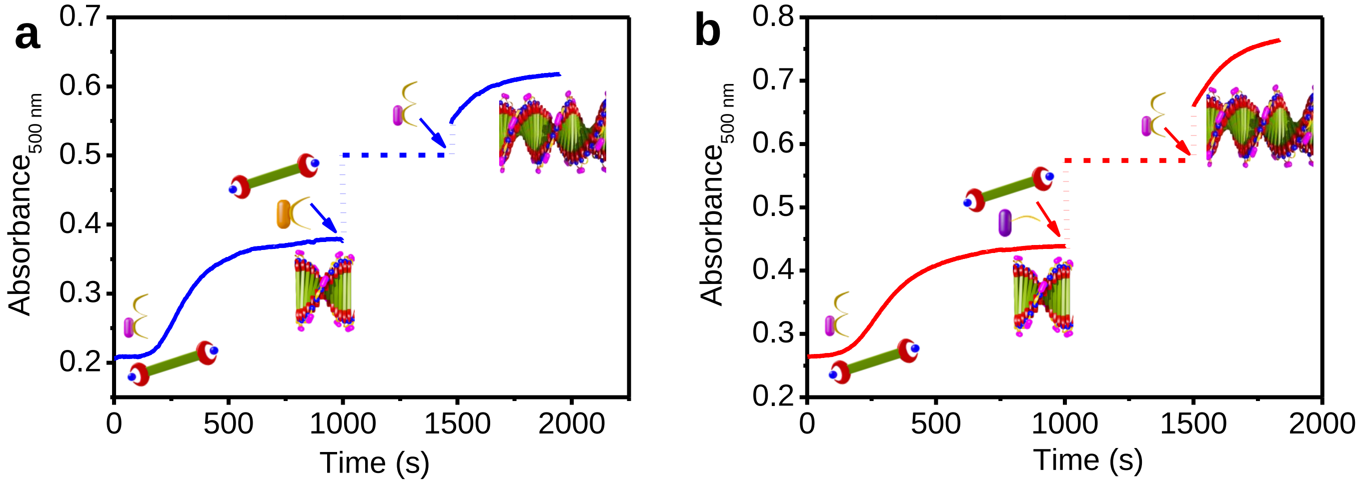

Supplementary Figure 28| Seeding with AMP and ADP on ATP-grown seed. Absorbance monitored at 500 nm showing no elongation on addition of **a**, 5 equiv. of AMP and **b**, 2 equiv. of ADP along with fresh feed of **1**, to a pre-grown ATP-**1** seed (dashed lines). However, the assembly starts growing on the seed as soon as 0.9 equiv. of ATP is introduced into the system as evident from the increase in absorbance ([S] = $2x10^{-5}$ M, [**1**] = $1x10^{-5}$ M (10 μL) CH_3CN/HEPES, 10/90, *v/v*, 30 ºC). Corresponding CD changes are shown in Fig. 4c.

Note: As evident from the CD studies as well, it is clear that both AMP and ADP are unable to induce elongation on ATP bound seeds. However an immediate increase observed in absorbance on addition of [***1***], corresponds to the increase in optical density due to overall increase in the concentration of **1** in solution. But there is an increase in the signal over time on addition of ATP to the solution indicating assembly of fresh feed on interaction with ATP on the existing seed due to competitive replacement of ADP and AMP by ATP.

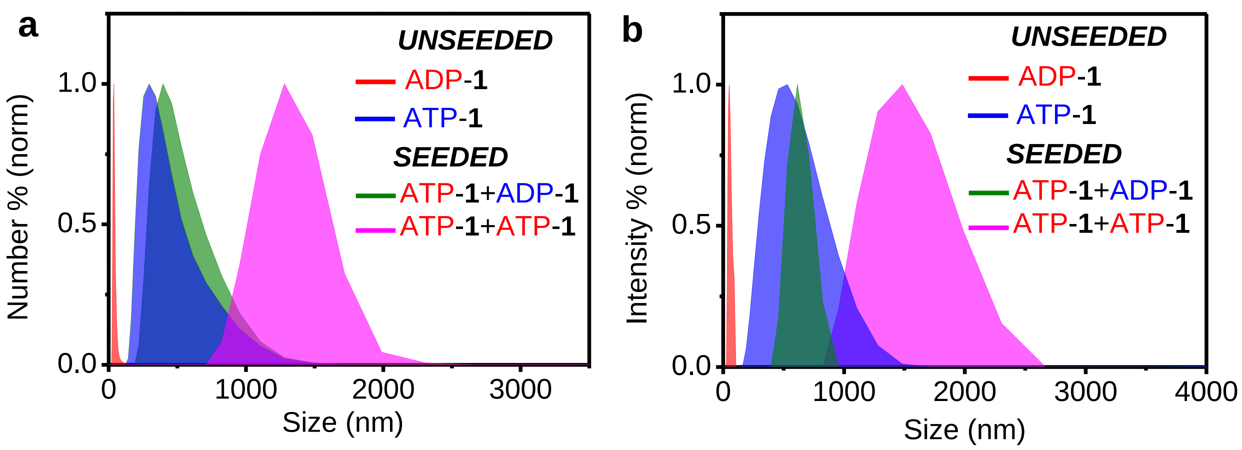

Supplementary Figure 29| DLS proof for ATP-selective seeded growth. Sizes got from **a**, number % and **b**, intensity % of ATP (blue) and ADP (red) bound **1** along with their seeded assembly with ATP (pink) and ADP (green) with fresh feed. These evidences indicate that ADP is unable to induce elongation on ATP bound seed. But ADP-**1** gives very small aggregates indicating that ADP does bind to **1** to give very short aggregates but is unable to elongate further in seeded or non-seeded manner. ([S] = 2x10^{-5} M, [**1**] = 2x10^{-5} M (10 μL), CH_3CN/HEPES, 10/90, *v/v*, 0.9 equiv. ATP, 2 equiv. ADP, 30 ºC).

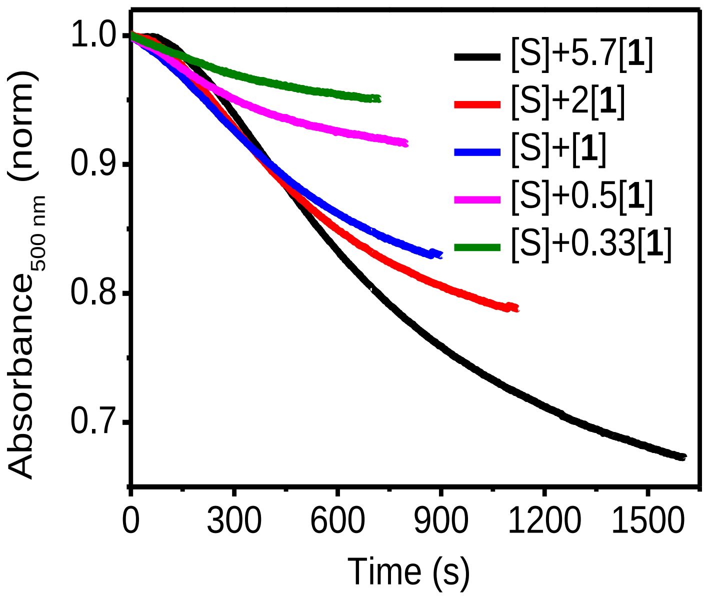

Supplementary Figure 30| Kinetics of the seeded growth. Normalised absorbance at time t = 0 s shows the consumption of fresh feed over time. With increasing concentration of monomer added to 1x10^{-5} M seed ([***1***]/[S] ratios of 0.33:1, 0.5:1, 1:1, 2:1 and 5.7:1), it takes a longer time for the completion of elongation with increase in rate of elongation. When 5.7x10^{-5} M of **1** is added to the seed, it gives rise to a sigmoidal absorbance change indicating the occurrence of some independent nucleation. ([S] = 1x10^{-5} M, [**1**] = 0.33x10^{-5} M (1.65 μL), 0.5x10^{-5} M (2.5 μL), 1x10^{-5} M (5 μL), 2x10^{-5} M (10 μL), 5.7x10^{-5} M (2.85 μL),CH_3CN/HEPES, 10/90, *v/v*, 0.9 equiv. ATP, 30 ºC).

Supplementary Table 4| Saturation time for seeding. Saturation time obtained from time-dependent CD intensity monitored at 450 nm on seeded growth for various [**1**]/[S] ratios of 0.33:1, 0.5:1, 1:1, 2:1 and 5.7:1 as shown in Fig. 4d of main text. It is evident that, with increasing concentration of [**1**], the time required for saturation increases because for a constant concentration of seed, higher concentration of monomers will take longer time to grow completely ([S] = $1x10^{-5}$ M, CH_3CN/HEPES, 10/90, *v/v*, 0.9 equiv. ATP, 30 ºC).

**S+xM**	t_{50} **(s)**	**Time taken for saturation (s)**
x=0.33	233 ± 2	728 ± 2
x=0.5	253 ± 3	805 ± 3
x=1	343 ± 1	900 ± 1
x=2	411 ± 3	1127 ± 3
x=5.7	588 ± 5	1611 ± 5

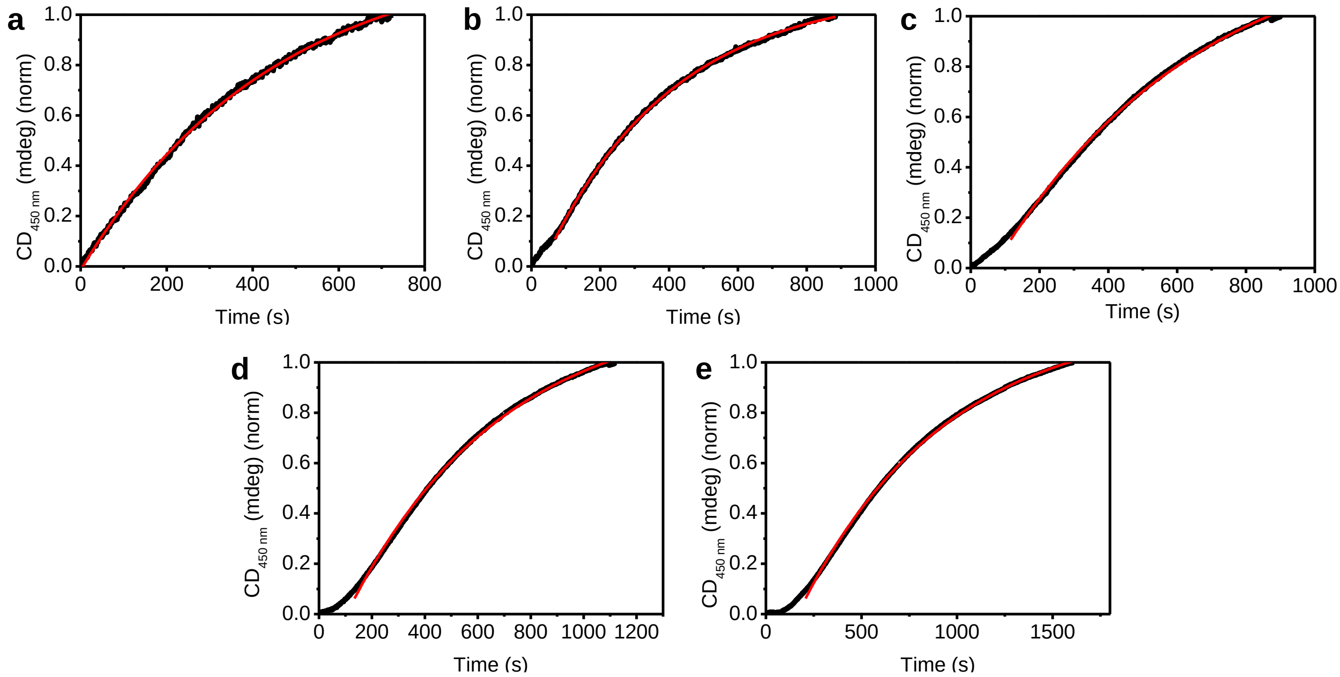

Supplementary Figure 31| Fitting of seeded growth kinetics. Seeded elongation kinetics curves fitted to obtain the corresponding *k* (rate constant for elongation). Seed conc. [S] = 1×10^{-5} M where [**1**] was varied as **a**, 0.33×10^{-5} M, **b**, 0.5×10^{-5} M, **c**,1×10^{-5} M, **d**, 2×10^{-5} M and **e**, 5.7×10^{-5} M.

The seeded elongation for different concentration of fresh feed was fitted by the well-known equation for seeded polymerization

$$[M]-[M]_{\infty} = ([M]_{o}-[M]_{\infty}) \exp(-k_{e(seeded)}[S]t) \tag{3}$$

which has been simplified from Oosawa's model[10] for actin polymerization by Moore (Supplementary Equation 3).[11] In the above experiments $[M]_o$ is the concentration of fresh feed added to a particular amount of seed [S]. $[M]_\infty$ corresponds to concentration of monomers coexisting with supramolecular polymer at equilibrium. [M] corresponds to the concentration of monomers at any time t elongating with a rate constant of $k_{e(seeded)}$. This equation could be written in a simplified manner:

$$y = a+b \exp(-k't) \tag{4}$$

where y = [M], a = $[M]_\infty$, b = $[M]_o-[M]_\infty$ and $k' = k_{e(seeded)}[S]$.

.

Supplementary Table 5| Kinetic parameters derived from the fitted data of seeded assembly. With increasing concentration of [**1**] added to 1x10^{-5} M of seed, there is a decrease in the value of *k'* and hence $k_{e(seeded)}$ by fitting to Supplementary Equation 4. This decrease indicates faster elongation with decrease of [**1**]. It may also be the result of some independent nucleation arising with increasing [**1**]/[S] ratio.

**[S]+x[1]**	**M_o (x10^{-5} M)**	***k'* (s^{-1} M)**	**$k_{e(seeded)}$ (s^{-1})**	**Adj. R^2**
x=0.33	0.33	2.4x10^{-5} ± 0.9x10^{-6}	2.4 ± 0.1	>0.99
x=0.5	0.5	2.23x10^{-5} ± 2.8x10^{-6}	2.23 ± 0.04	>0.99
x=1	1	1.68x10^{-5} ± 1.0x10^{-6}	1.68 ± 0.08	>0.99
x=2	2	1.64x10^{-5} ± 2.1x10^{-6}	1.64 ± 0.15	>0.99
x=5.7	5.7	1.3x10^{-5} ± 1.5x10^{-6}	1.3 ± 0.11	>0.99

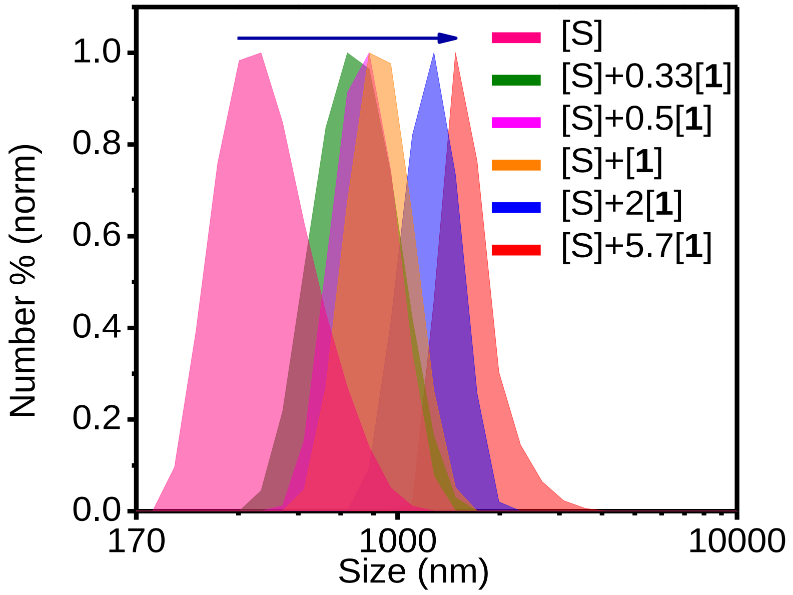

Supplementary Figure 32| Controlling the size of APT-1 assembly by seeding. Number % DLS data showing the increase in hydrodynamic radii from seed (S) to varying [**1**]/[S] ratios. Corresponding Intensity % DLS data has been shown in Fig. 4f. ([S] = 1x10^{-5} M, [**1**] = 1x10^{-5} M) (CH_3CN/HEPES, 10/90, *v/v*, 0.9 equiv. ATP, 30 ^oC).

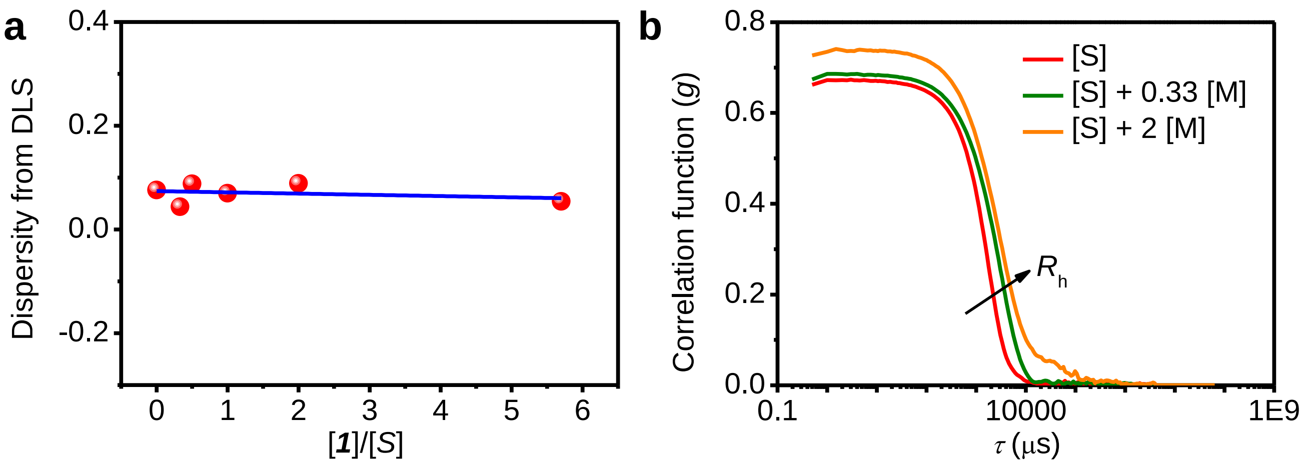

Supplementary Figure 33| Parameters derived from DLS of seeding experiments. Dispersity value derived directly from DLS with an average value calculated as 0.07 showing a narrow polydisperisty of the system. Dispersity values remain the same during the seeding experiments. **b,** Correlation function change for unseeded (black curve) and seeded (green for [**1**]/[S] = 0.33 and orange for [**1**]/[S] = 2) data shows an increase in lag time on seeding showing an increase in size with increase in scattering on seeding. Dispersity trends obtained directly from DLS shows a controlled supramolecular polymerization reaction with good quality correlation curves from DLS.

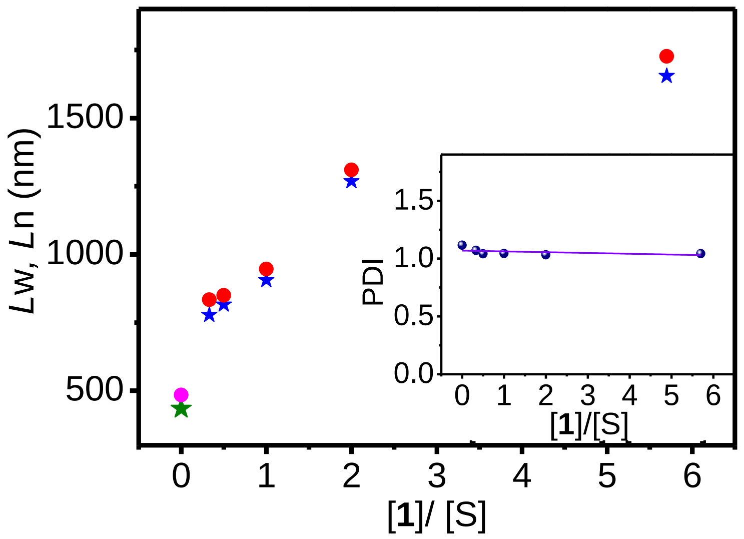

Supplementary Figure 34| Parameters calculated from DLS of seeding experiments. Weight-average length (*L*w) (star, green = [S], blue = [S]+x[**1**]), number-average length (*L*n) (filled circles, pink = [S], red = [S]+x[**1**]) . Inset shows the PDI (L*w*/L*n*) as a function of the ratio of the total amount of added fresh feed to the initial amount of seed. ([S] = 1x10^{-5} M, [**1**] = 1x10^{-5} M, CH_3CN/HEPES, 10/90, *v/v*, 0.9 equiv. ATP, 30 ^{o}C). These data indicate that both *L*w and *L*n follow a similar linear trend in increase with *increasing [**1**]/[S]*

ratio. The polydispersity index (PDI) of the system also remains constant with an average value of 1.1 suggesting a good control over the degree of polymerization and dispersity.

Though the *L*w and *L*n values obtained from DLS distribution is not conventional for anisotropic structures, we would like to specify that we are interested only in the trends obtained and not the absolute values and hence presented the data in Supplementary Fig. 34. In addition the fibres generated in solution are actually not of high aspect ratio as evidence from smaller fibres from TEM. Thus we believe that fibres in solution are still small enough to fall under a rough assessment of size by DLS. Although we have tried the SLS experiments, but unfortunately due to a combination of factors (long time molecular stability of sample in solution as the time taken for multi angle SLS is more, lack of strong scattering) these experiments did not fructify.

$$L\mathrm{n} = \frac{\sum_{i=1}^{n} NiLi}{\sum_{i=1}^{n} Ni} \qquad L\mathrm{w} = \frac{\sum_{i=1}^{n} NiLi^2}{\sum_{i=1}^{n} NiLi} \qquad \mathrm{PDI} = \frac{L\mathrm{w}}{L\mathrm{n}} \tag{5}$$

Number average chain length (*L*n), weight average chain length (*L*w) and polydispersity index (PDI) were calculated from the DLS data for different concentrations of fresh feed. Here *n* corresponds to the total number of points got for all the DLS data. *Li* represents the mean length of size *i* and *Ni* corresponds to the fraction of total number of self-assembled structures of the corresponding size.

Dispersity values obtained directly from DLS as well as calculated from length statistics shows a controlled supramolecular polymerization reaction with good quality correlation curves from DLS.

Enzyme mediated transient self-assembly

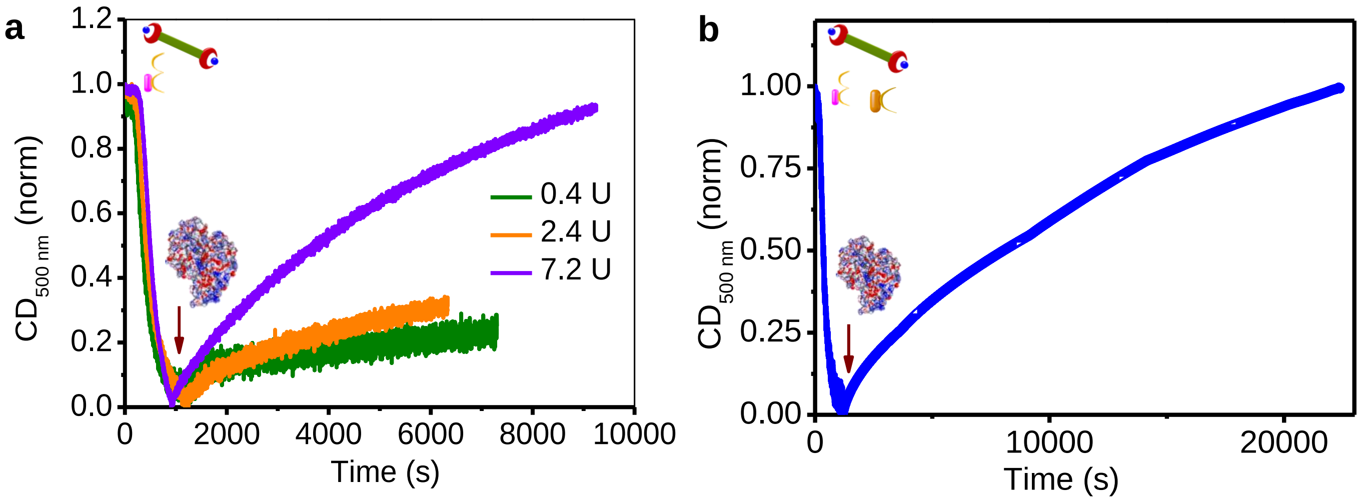

Supplementary Figure 35| Passive assembly of ATP-1. CD spectral changes of **a**, ATP-**1** grown stacks on introducing various amounts of the enzyme potato apyrase and **b**, ATP-**1** grown stacks in the presence of 0.9 equiv. of ADP on introducing 7.2 units of the enzyme potato apyrase, monitored at 500 nm. ([**1**] = 2x10^{-5} M, CH_3CN/HEPES, 10/90, *v/v*, 0.9 equiv. ATP, 0.9 equiv. of ADP, 30 ºC). The decrease in CD signal with time is clear indication of the dis-assembly of the stacks because of the hydrolysis of ATP. The rate of disassembly became faster on increasing the enzyme concentration, clearly indicating an enzyme controlled process. To understand if enzyme selectively hydrolyses the unbound or bound phosphates, CD kinetic measurements of ATP-**1** in presence of other phosphates (0.9 equiv. of ATP followed by addition of 0.9 equiv. of ADP) were performed. The decrease in CD signal with time is clear indication of the dis-assembly of the stacks because of the hydrolysis of ATP. As expected, due to competitive binding, ATP should be bound to molecule **1** to form ATP-**1** stacks, whereas ADP must be free in solution. Gradual decrease in signal immediately after completion of elongation without a lag, suggests that apyrase has no preferential action to unbound phosphates compared to bound ones. If the enzyme were to preferentially act on unbound phosphates, a constant CD signal would have been obtained initially till all unbound phosphates are consumed and only then signal should have started to decrease. These results suggest that the enzyme acts on the ATP bound on to the stacks though we do not rule out an exchange of nucleotides, as we have already shown a fast nucleotide exchange on similar system in our previous studies, using competitive gust binding experiments.

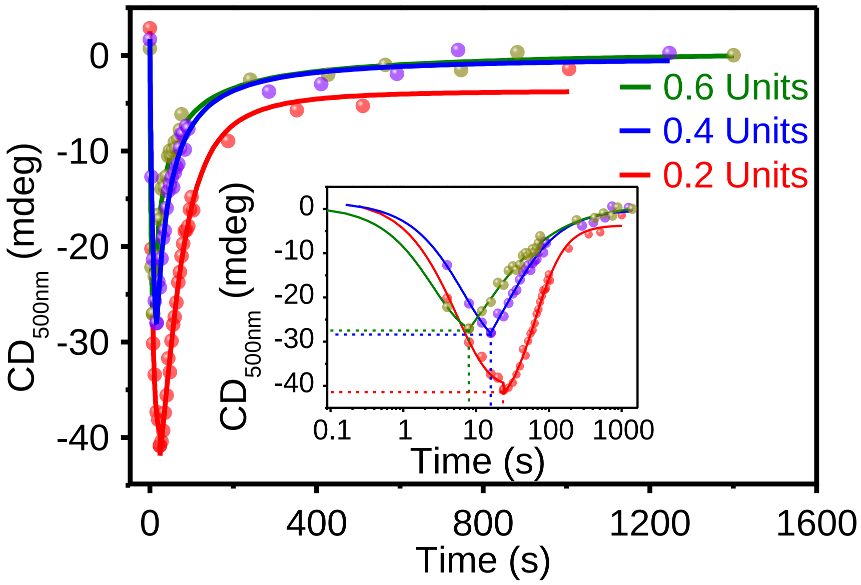

Supplementary Figure 36| Active assembly of ATP-1. Transient assembly of **1** in presence of 6.4 equiv of ATP and apyrase as evident from the time-dependent CD changes at 500 nm, which showed a growth followed by the disassembly. A higher equiv. of ATP is required for inducing an assembly in presence of enzyme due to competitive hydrolysis of unbound ATP. Before required equiv. of ATP is able to bind with **1** to induce elongation, it gets hydrolysed, hence no elongation seen with 0.9 equiv. of ATP in the presence of apyrase. ([**1**] = $2x10^{-5}$ M, CH_3CN/HEPES, 10/90, *v/v*, 6.4 equiv. ATP, 30 ºC)

Inset shows the zoomed portion of the transient phase in the log scale to clearly show the changes observed for varying units of enzyme. 0.6 (green), 0.4 (red) and 0.2 (blue) units of potato apyrase has been used for the studies.

Supplementary Table 6| Parameters derived from the transient experiments.

Enzyme Units (U/mL)	Frequency s^{-1}	Amplitude (mdeg)
0.2	$12.5x10^{-2}$	41
0.4	$6.25x10^{-2}$	29
0.6	$4.17x10^{-2}$	27

These experiments show that with a higher unit of apyrase, there is faster hydrolysis of ATP hence giving rise to faster disassembly as seen from faster decay in CD signal. In presence of 0.6 units of apyrase the extent of elongation is also less as compared to 0.2 units of apyrase as seen from the frequency and amplitudes calculated from the CD spectra. This is observed due to the presence of higher units of enzyme, the hydrolysis supersedes elongation faster than for a lower unit of enzyme.

Results from Computations and MM/MD simulations:

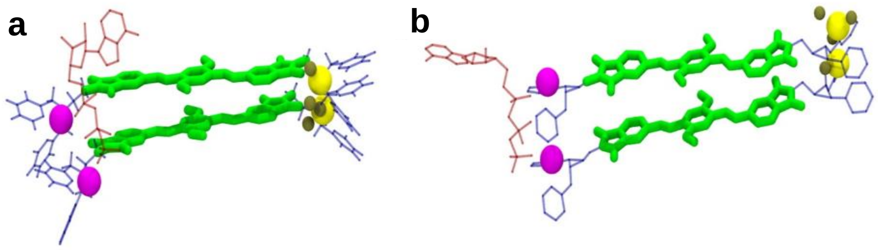

Supplementary Figure 37| Gas phase DFT calculations of the ATP-1 dimer. **a,** Initial and **b,** final configurations of ATP-**1** dimer. Colour Scheme: Green - OPV, Blue - DPA, Red - ATP/GTP. Zinc atoms are represented as spheres in two different colours (yellow and magenta) to emphasize helicity and the counter ions (Cl^-) shown in tan colour.

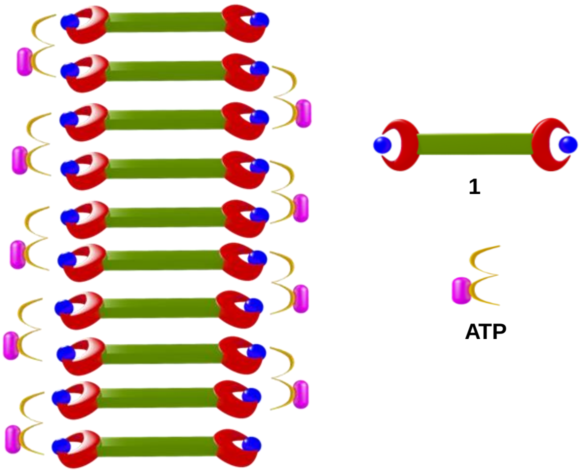

Supplementary Figure 38| Schematic model of ATP-**1** employed in MM/MD simulations.

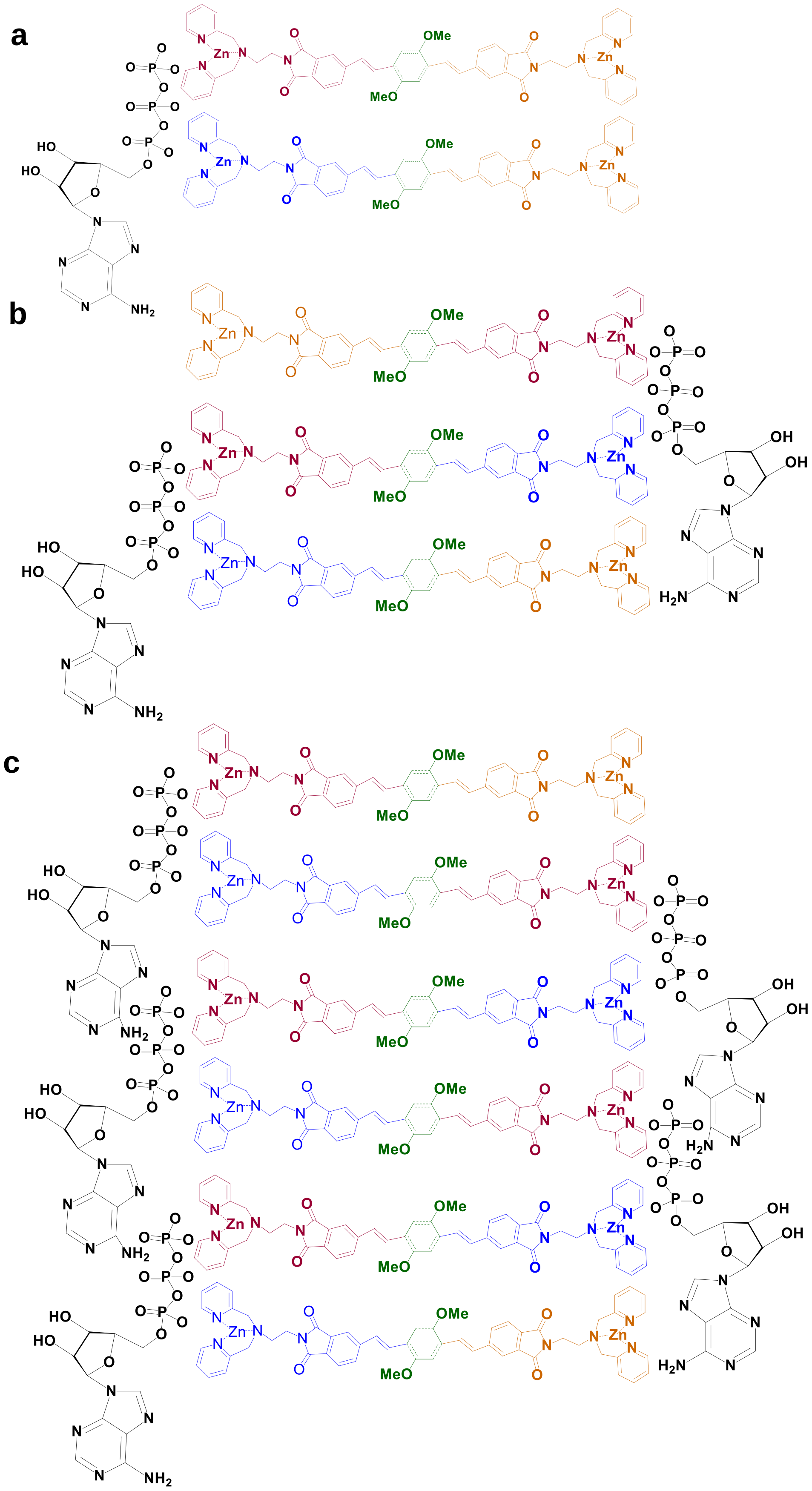

Supplementary Figure 39| Segment-wise representation of ATP-1 oligomers. a, Dimer, **b,** trimer and **c,** hexamer. Segments with the same colour code have the same atomic site charges across different oligomers. The site charges themselves are determined for the dimer via gas phase DFT calculations.

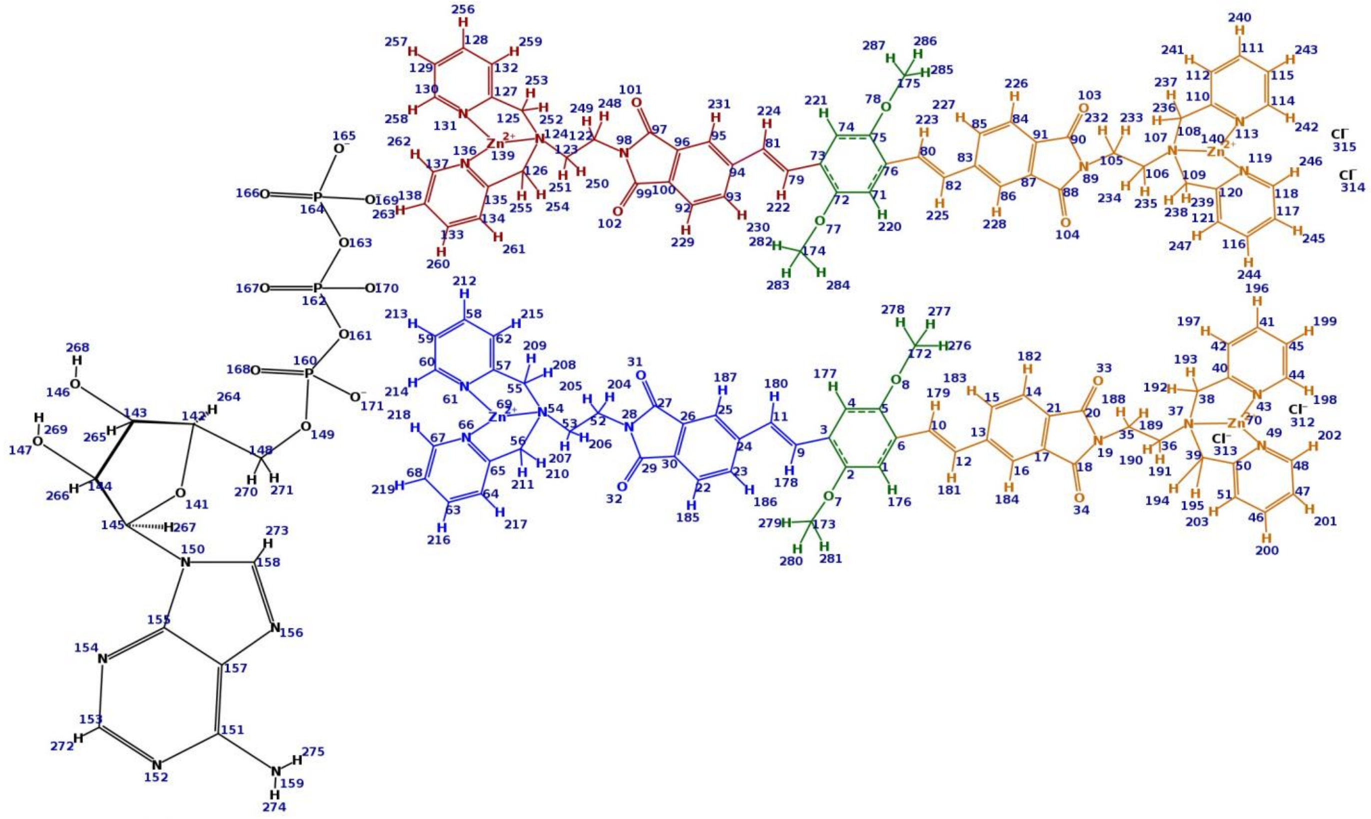

Supplementary Figure 40| Atom mapping scheme in the ATP-**1** dimer.

Supplementary Table 7| Partial charges on atoms coloured green (Segment 1).

Atom Index	Charge (e)
1	0.2001260
2	-0.2822740
3	0.0245920
4	0.2152150
5	-0.2780810
6	0.0378690
7	0.1232810
8	0.1331140
172	0.4697920
173	0.4752950
276	0.3735670
277	0.3724460
278	0.1301210
279	0.0718660
280	0.3300760
281	0.3962770

Supplementary Table 8| Partial charges on atoms coloured blue (Segment 2).

Atom Index	Charge (e)
9	-0.2266090
11	-0.1069550
23	-0.1495710
24	0.1124240
25	-0.1623110
26	0.1044030
27	0.0991130
28	-0.1166080
29	-0.1371350
30	-0.0875030
31	0.1357640
32	-0.0604140
52	-0.4596130
53	-0.4241250
54	-0.2513020
55	-0.0327050
56	0.1005830
57	0.0947900

58	-0.1192450
59	0.0821550
60	0.1120950
61	-0.0661520
62	-0.1720780
63	0.1302860
64	0.0969930
65	-0.1779110
66	0.0911470
67	0.1311690
68	0.2510930
69	-0.2545910
178	0.1139980
180	-0.0710940
185	0.1159320
186	0.1088430
187	-0.1589540
204	-0.2143040
205	0.1001020
206	0.1099340
207	0.1451300

208	-0.2895450
209	-0.2902530
210	-0.0505850
211	-0.0460950
212	0.1126610
213	0.0797540
214	0.0565160
215	-0.1599560
216	-0.1746060
217	-0.1557470
218	0.1549310
219	0.0836320

Supplementary Table 9| Partial charges on atoms coloured brown (Segment 3).

Atom Index	Charge (e)
79	0.1207740
81	0.1389940
92	0.0817870
93	-0.2580400
94	-0.0616500
95	-0.1170080
96	0.0764660
97	0.0907790
98	0.2525670
99	0.0919510
100	0.1242300
101	0.2771600
102	-0.2398790
122	0.8612990
123	0.2186420
124	-0.2735670
125	0.0221610
126	0.2122020
127	-0.2968420
128	0.0323720

129	0.1224910
130	0.1260280
131	-0.2272900
132	-0.2285050
133	-0.0951440
134	0.1120180
135	0.0799680
136	0.0894010
137	-0.1068700
138	0.0997350
139	0.1155400
222	0.1219070
224	-0.2251400
229	0.1150890
230	0.1696170
231	0.0013040
248	-0.5495240
249	0.0223980
250	0.1608390
251	-0.3487000

252	0.2304780
253	-0.0958370
254	0.1642070
255	-0.4311570
256	-0.0399050
257	0.5057720
258	-0.7468710
259	-0.5403430
260	0.3320840
261	-0.4552300
262	0.2389500
263	0.1360720

Supplementary Table 10| Partial charges on atoms coloured orange (Segment 4).

Atom Index	Charge (e)
10	-0.2268170
12	0.1124760
13	0.0949340
14	0.0836820
15	-0.1052880
16	0.0797580
17	0.1136890
18	0.0944590
19	-0.1022510
20	0.1112070
21	-0.0942390
35	0.1333100
36	0.1064510
37	-0.1168010
38	-0.1477280
39	-0.0743680
40	0.1436270
41	-0.0629300
42	0.1201170
43	-0.1210500

44	0.1315040
45	0.4780740
46	0.4716750
47	-0.4204660
48	-0.4554930
49	-0.2208780
50	0.4761820
51	0.4783470
179	0.1074420
181	0.1025960
182	0.0717880
183	-0.1122140
184	-0.1793490
188	0.0923560
189	0.1268300
190	0.2632420
191	-0.2086070
192	0.1243500
193	0.0078360
194	0.1192220

195	-0.2135850
196	0.1120080
197	0.1115810
198	0.1393250
199	0.2666950
200	-0.2491500
201	0.1404980
202	0.0008720
203	0.1438200

Supplementary Table 11| Partial charges on atoms coloured black (Segment 5).

**Atom Index**	**Charge (e)**
141	-0.1094850
142	0.1222890
143	-0.0810360
144	0.1164130
145	-0.1572180
146	0.1035350
147	-0.1763640
148	0.1158830
149	0.0935130

150	-0.1244410
151	-0.1526800
152	-0.0798950
153	0.1396250
154	-0.0715840
155	0.1166360
156	-0.1019970
157	0.1390820
158	0.1161540
159	-0.1198090
160	-0.1614720
161	-0.0814980
162	0.1366790
163	-0.0596450
164	0.1230850
165	-0.1186920
166	0.1313450
167	0.4713770
168	0.4650240
169	-0.4359130
170	-0.4310800

171	-0.2107810
172	0.4697920
264	0.1005910
265	-0.5511750
266	-0.5628490
267	-0.6710930
268	1.5806230
269	-0.6466810
270	1.5442340
271	-0.8383400
272	-0.7795030
273	-0.7676080
274	-0.8010060
275	-0.8466240

Supplementary Table 12| Partial charges on counter ion (Cl^-)

**Atom index**	**Charge (e-)**
288	-0.4678910
289	-0.4933320
290	-0.5463590
291	-0.5266740

Supplementary Table 13| System information over the oligomers for MM/MD simulations

Size of Oligomer (n)	Number of '1'	Number of ATPs	Number of Counterions (Cl^-)	Number of Solvent molecules (H_2O)	Box dimensions (Å)
2	2	1	4	6075	(60,60,60)
3	3	2	4	5932	(60,60,60)
4	4	3	4	5765	(60,60,60)
6	6	5	6	5448	(60,60,60)
10	10	9	4	4929	(60,60,60)
15	15	14	4	5295	(60,60,80)
25	25	24	9	18551	(66.88,66.88,140.6)

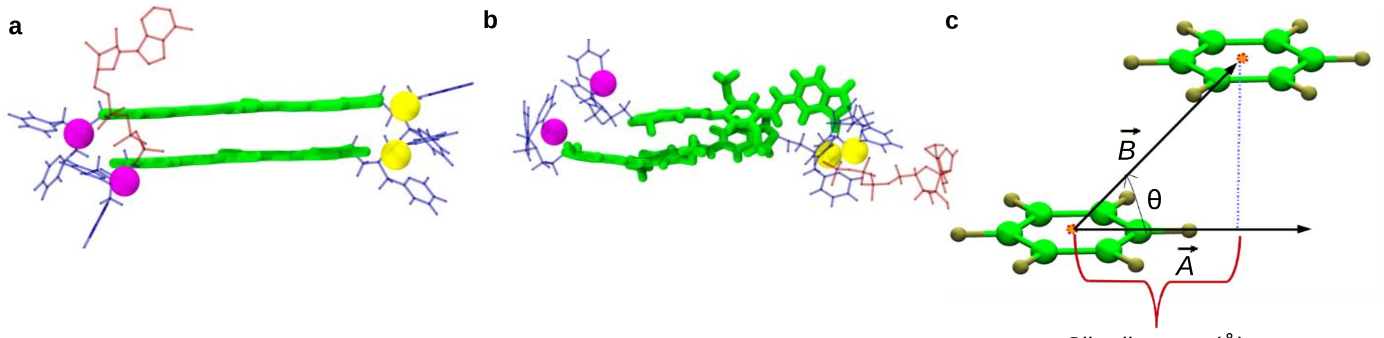

Supplementary Figure 41| Simulated Dimer. a, Initial configuration of the dimer. **b,** Final snapshot of the dimer from MM/MD simulation. **c,** Definition of slip distance (Supplementary Equation 5) (Colour scheme is same as in Supplementary Fig. 33).

Note: A simulation of a dimer with initial structure as shown in Supplementary Fig. 41 a was performed. The final snapshot of the dimer after 30 ns is shown in Supplementary Fig. 41 b. In a dimer, the ends of molecule **1** which are not bound to ATPs are fluxional. In order to gain donor-acceptor interaction, molecule **1** slide over another. To quantify the magnitude of this slide, a slip distance defined as the lateral displacement of the central moiety in molecule **1** as shown Supplementary Fig. 41 c is employed.

$$\text{Slip distance} = |\vec{\boldsymbol{A}}||\vec{\boldsymbol{B}}|\cos\theta \qquad (5)$$

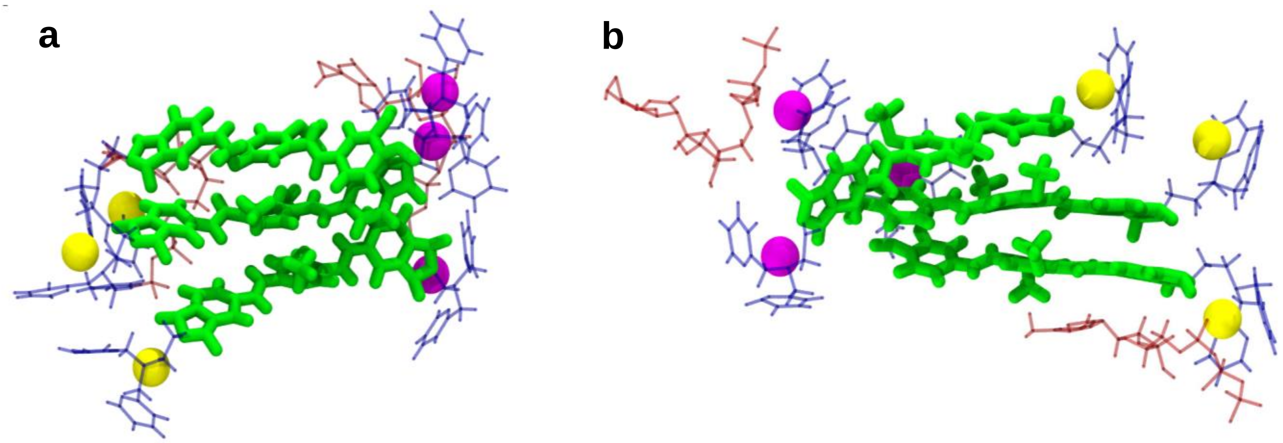

Supplementary Figure 42| Simulated Trimer. a, Initial structure of the preformed trimer bound to ATP on both sides. **b,** Snapshot of the trimer after 30 ns at 298.15K. The slip distance in the trimer is 3.55 Å, smaller than the value of 5.2 Å (Supplementary Equation 5) seen in the dimer.

Note: A tetramer was also modelled in solution at 298.15 K (Fig. 2g). The final snapshot of the tetramer are shown in Supplementary Fig. 18a. Simulations of hexamer, decamer, pentadecamer and icosikaipentamer were also similarly performed, and their final snapshots are shown in Supplementary Fig. 18 b, c, d and Fig. 2g. In the longer oligomers, the molecules are well ordered through intermolecular hydrogen bonds between the ATPs. The increase in the number of ATPs makes the assembly more rigid. The interaction between the ATPs enhances the chirality of the stack and a low slip value of 1.35 Å was observed. With increasing oligomer size, chirality is enhanced and the slip distance decreases. The normalized distribution of slip distance is shown in Fig. 2e.

Supplementary Table 14| Slip distances calculated for various oligomers. Slip distances from MM/MD simulations calculated using (Supplementary Equation 5).

Oligomer size	Slip distance (Å)
Dimer	5.20
Trimer	3.55
Tetramer	4.42
Hexamer	3.25
Decamer	2.07
Pentadecamer	2.37
Icosikaipentamer	1.35

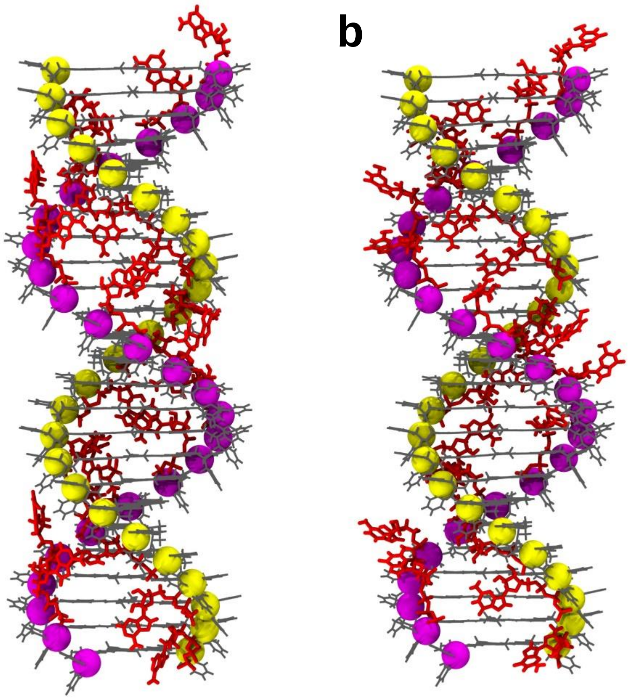

Supplementary Figure 43| Structures of preformed icosikaipentamer in MM/MD simulations. Snapshots of initial structures of preformed **a**, ATP-**1** and **b**, GTP-**1** 25-mer. Zinc atoms have been depicted as spheres of two different colours (magenta and yellow) to demonstrate the helicity in the oligomers of the initial configurations in either of the stacks. Molecules of **1** are depicted in black sticks (in a and b) and ATP and GTP have been shown in red. As described in the main text, although both systems are initiated from a highly ordered helical arrangement, the ATP-**1** stack retains it, while the GTP-**1** stack exhibits considerable disorder during the MM/MD simulation trajectory.

Supplementary References:

1. Norman, M. H., Kelley, J. L. & Hollingsworth, E. B. Conformationally restricted analogues of remoxipride as potential antipsychotic agents. *J. Med. Chem.* **36**, 3417-3423 (1993).

2. Lee, H. N. *et al*. Pyrophosphate-selective fluorescent chemosensor at physiological pH: Formation of a unique excimer upon addition of pyrophosphate. *J. Am. Chem. Soc.* **129**, 3828-3829 (2007).

3. Grudzień, K., Malinska, M. & Barbasiewicz, M. Synthesis and properties of bimetallic Hoveyda-Grubbs metathesis catalysts. *Organometallics* **31**, 3636-3646 (2012).

4. Dautel, O. J. *et al*. Nanostructuration of phenylenevinylenediimide-bridged silsesquioxane: From electroluminescent molecular J-Aggregates to photoresponsive polymeric H-aggregates. *J. Am. Chem. Soc.* **128**, 4892-4901 (2006).

5. Delbosc, N. *et al*. Control of the aggregation of a phenylenevinylenediimide chromophore by use of supramolecular chemistry: Enhanced electroluminescence in supramolecular organic devices. *Chem. Mater*. **22**, 5258-5270 (2010).

6. Shoffnera, S. K. & Schnell, S. Estimation of the lag time in a subsequent monomer addition model for fibril elongation. *Phys. Chem. Chem. Phys.* **18**, 21259-21268 (2016).

7. Morris, A. M., Watzky, M. A. & Finke, R. G. Protein aggregation kinetics, mechanism, and curve-fitting: A review of the literature. *Biochim. Biophys. Acta* **1794**, 375-397 (2009).

8. Ojida, A., Mito-oka, Y., Sada, K. & Hamachi, I. Molecular recognition and fluorescence sensing of monophosphorylated peptides in aqueous solution by bis(zinc(II)-dipicolylamine)-based artificial receptors. *J. Am. Chem. Soc.* **126**, 2454-2463 (2004).

9. Ojida, A., Takashima, I., Kohira, T., Nonaka, H. & Hamachi, I. Turn-on fluorescence sensing of nucleoside polyphosphates using a xanthene-based Zn(II) complex chemosensor. *J. Am. Chem. Soc.* **130**, 12095-12101 (2008).

10. Oosawa, F. & Asakura, S. Thermodynamics of the polymerization of protein. *Academic Press Inc., New York* (1975).

11. Zhao, D. & Moore, J. S. Nucleation–elongation: a mechanism for cooperative supramolecular polymerization. *Org. Biomol. Chem*. **1**, 3471-3491 (2003).

12. Kumar, M., Brocorens, P., Tonnelé, C., Beljonne, D., Surin, M. & George, S. J. Dynamic supramolecular polymer with stimuli responsive handedness for in situ probing of enzymatic ATP hydrolysis. *Nat. Commun.* **5**, 5793 (2014)

.
